# Arf6-driven endocytic recycling of CD147 determines HCC malignant phenotypes

**DOI:** 10.1186/s13046-019-1464-9

**Published:** 2019-11-21

**Authors:** Shanshan Qi, Linjia Su, Jing Li, Chuanshan Zhang, Zhe Ma, Guiqiu Liu, Qing Zhang, Guhe Jia, Yongjun Piao, Sihe Zhang

**Affiliations:** 1 Department of Cell Biology, School of Medicine, Nankai University, 94 Weijin Road, Nankai District, Tianjin, 300071 People’s Republic of China; 2 Department of Pathology, Third Central Hospital of Tianjin Medical University, 83 Jintang Road, Tianjin, 300170 China; 3 Department of Clinical Laboratory, Cancer Hospital of Tianjin Medical University, Huan Hu Xi Road, Ti Yuan Bei, He Xi District, Tianjin, 300060 China

**Keywords:** Arf6, CD147, Endocytic recycling, Malignant phenotype, Liver cancer

## Abstract

**Background:**

Adhesion molecules distributed on the cell-surface depends upon their dynamic trafficking that plays an important role during cancer progression. ADP-ribosylation factor 6 (Arf6) is a master regulator of membrane trafficking. CD147, a tumor-related adhesive protein, can promote the invasion of liver cancer. However, the role of Arf6 in CD147 trafficking and its contribution to liver cancer progression remain unclear.

**Methods:**

Stable liver cancer cell lines with Arf6 silencing and over-expression were established. Confocal imaging, flow cytometry, biotinylation and endomembrane isolation were used to detect CD147 uptake and recycling. GST-pull down, gelatin zymography, immunofluorescence, cell adhesion, aggregation and tight junction formation, Transwell migration, and invasion assays were used to examine the cellular phenotypes. GEPIA bioinformatics, patient’s specimens and electronic records collection, and immunohistochemistry were performed to obtain the clinical relevance for Arf6-CD147 signaling.

**Results:**

We found that the endocytic recycling of CD147 in liver cancer cells was controlled by Arf6 through concurrent Rab5 and Rab22 activation. Disruption of Arf6-mediated CD147 trafficking reduced the cell-matrix and cell-cell adhesion, weakened cell aggregation and junction stability, attenuated MMPs secretion and cytoskeleton reorganization, impaired HGF-stimulated Rac1 activation, and markedly decreased the migration and invasion of liver cancer cells. Moreover, high-expression of the Arf6-CD147 signaling components in HCC (hepatocellular carcinoma) was closely correlated with poor clinical outcome of patients.

**Conclusions:**

Our results revealed that Arf6-mediated CD147 endocytic recycling is required for the malignant phenotypes of liver cancer. The Arf6-driven signaling machinery provides excellent biomarkers or therapeutic targets for the prevention of liver cancer.

## Background

Dynamic endocytosis and recycling of membrane proteins control numerous pathophysiological functions including cell homeostasis, nutrient uptake, and oncogenic signaling. Membrane proteins containing the clathrin-adapter-bound sequence are internalized via clathrin-mediated endocytosis (CME). When they lack specific sorting sequence they enter the cell through clathrin-independent endocytosis (CIE). Two types of CIE cargo proteins join different trafficking itineraries within the cell [1]. One (such as MHCI, CD59, and CD55) travels along a bulk route (B-cargo) that directs some cargos for slow recycling and some cargos toward lysosomes for degradation. Another (including CD147, CD98, CD44, and Glut1) travels an alternative route (A-cargo) in which nearly all of these cargos are rapidly recycled to the cell surface [2]. As a consequence, CD147 and other ‘A-cargo’ proteins are long-lived.

CD147 (Basigin/EMMPRIN) is an adhesion molecule overexpressed in multiple tumors [3, 4]. Mature CD147 is an N-linked glycosylated protein and exists both in transmembrane and soluble forms. Depending on the glycosylation level, CD147 exists in two forms: high-glycosylated CD147 (HG-CD147) and low-glycosylated CD147 (LG-CD147) [5, 6]. Because it is activity involved in metabolic reprogramming, apoptosis inhibition, motile migration, and multidrug resistance, CD147 serves as a hub protein in hepatocarcinogenesis [5, 7–9]. Although previous studies revealed the role of CD147 trafficking in the progression of several types of cancers [10–12], the detailed regulation signal in liver cancer needs to be clarified.

Cell-surface protein abundance is controlled by G protein (guanine nucleotide-binding protein)-mediated transport through the regulation of endocytic recycling. Previous studies reported that CD147 is internalized through Rab5-associated CIE, recycled via Rab22-dependent endosomes, and by-passed merging with the EEA1-positive endosomes in cervical cancer cells [13–15]. Arf6 (ADP-ribosylation factor 6) is another GTPase that regulates the endocytic recycling process in concert with different Rab GTPases [2, 16–22]. Arf6 activation can further promote CD147 trafficking, especially to accelerate it entering in the fast recycling pathway [15, 23]. This might avoid its transport to the slow recycling route through Rab11-positive endosomes or the default degradation pathway. Although Arf6 and Rab GTPases are independently involved in the CD147 trafficking process [9, 10, 15, 24–27], investigations on the precise mechanism governing CD147 distribution on the cancer cell surface are still required.

In this study, the role of Arf6 on CD147 trafficking in liver cancer cells and its contribution to the malignant behaviors of HCC (hepatocellular carcinoma) were examined. We showed that, by modulating the Rab5 and Rab22 co-activation, cell adhesion and junction formation, Arf6-driven CD147 endocytic recycling causes liver cancer cells to acquire migratory and invasive phenotypes. The Arf6-mediated CD147 signaling functions as a critical determinant for poor clinical outcome of HCC patients.

## Material and methods

### Cell cultures, plasmids, antibodies, and chemicals

7721, HepG2, and Huh7 cells were obtained from Type Culture Collection of the Chinese Academy of Sciences (China). Arf6(wt)- and Arf6(Q67L)-HA/pcDNA3 plasmids were kindly supplied by Dr. Shumei Wei (Zhejiang University) [28]. EEA1- and Rabaptin-5/pGEX-4T-3 plasmids were kindly supplied by Prof. Byung-Ha Oh (Korea Advanced Institute of Science and Technology). The pGEX-GST-PAK (CRIB) plasmid was previously obtained from the Lab of Prof.  (Memorial Sloan-Kettering Cancer Center).

Mouse anti-CD147 Ab (H18) was originally produced [7]. Rabbit anti-Rab5 Ab (D160063), anti-Rab22 Ab (D160036), anti-Rac1 (SC-95), anti-Na/K ATPase Ab (sc-71,637) and mouse anti-Arf6 (sc-7971) were obtained from Santa Cruz. Rabbit anti-ZO-1 Ab (WL03419), anti-E-cadherin Ab (WL01482), anti-pan-cadherin Ab (WL03295) and anti-β-catenin Ab (WL0962a) were obtained from Wanleibio. Rabbit anti-CD147 PcAb (AB22048b), anti-HA Ab (D110004), mouse anti-β-actin Ab (D190606) and collagen (A001654) were obtained from Sangon Biotech. Mouse anti-ARNO Ab (AA 314–399) was obtained from 4A Biotech. Cell fractionation isolation kits (89881 and 78840), goat anti-mouse Ab-Alexa Fluor (AF) 488 (A-11001), anti-mouse AF647 (A-31571), anti-rabbit Ab-AF647 (A27040), NeutrAvidin agarose (29200), GSH Glutathione sepharose (G2879), Hochest33342 (62249), and Rhodamine-phalloidin (R415) were obtained from Life Technologies. Matrigel (356234), fibronectin (F2006), laminin (L2020), gelatin (G1890), polybrene (H9268), puromycin (P7255), NHS-SS-biotin (21328), HGF (H0536), and the remaining chemicals used in this study were from Sigma.

### Gene stable knock-down and overexpression

The pLV-RNAi system (BIOSETTIA, SORT-B19) was utilized to produce Arf6-KD (knocked-down) stable cell lines. Three independent Arf6-targeting shRNA (A1: AAAAGGAAGGTGCTATCCAAAATTTGGATCCAAATTTTGGATAGCACCTTCC, A2: AAAACAACAATCCTGTACAAGTTGATTGGATCCAATCAACTTGTACAGGATTGTTG, and A3: AAAAGCTCACATGGTTAACCTCTAATTGGATCCAATTAGAGGTTAACCATGTGAGC) were generated as previously described [29]. Arf6(wt) and Arf6(Q67L) plasmids were transfected in liver cancer cells for gene over-expression.

### Biotinylation and subcellular fractionation

Cell-surface and endomembrane proteins were isolated by using fractionation kits. Briefly, dish-grown confluent cells were surface-labeled with NHS-SS-biotin (0.2 mg/ml in PBS) at 4 °C for 30 min, quenched with 0.1 M glycine, and collected and solubilized by sonication. Cell lysates were applied on a NeutrAvidin agarose-loaded column, and the labeled proteins were centrifuge-eluted after incubation with 50 mM DTT solution. For endomembrane isolation, detached cells were digested with Typsin again (to maximize the removal of the surface protein). The cell pellet was incubated with cold cytoplasmic extraction buffer for 10 min, and the resulting pellet was further treated with cold membrane extraction buffer for 10 min. The final supernatant was the extracted endomembrane fraction. The resulting samples were corrected to equivalent protein concentrations and levels of CD147 (cell surface vs endomembrane fraction) as determined by Western blot.

### Antigen internalization and recycling

CD147 uptake was measured as previously described [9, 30]. Briefly, coverslip-grown cells were incubated with the H18Ab-AF488 complex (H18Ab, 1:500, anti-mouse AF488, 1:1000) for 15 min at 37 °C, rinsed with PBS/1 M NaCl, fixed with 4% PFA, permeabilized with 0.2% saponin, FBS-blocked and stained with anti-HA Ab plus anti-rabbit-AF647 at 4 °C overnight. Cells were imaged under a confocal microscope. Eight-bit maximal projections of the z-series were established using ImageJ software. To determine the uptake by flow cytometry, trypan blue was added to quench cell surface-associated fluorescence before cells were trypsinized for immediate analysis. Controls without H18Ab were included in all experiments. 80 cells were confocal-imaged in each group, and 10,000 cells per sample were counted by flow-cytometry.

CD147 recycling was performed as previous reported without biotinylation [9, 30]. Briefly, coverslip-grown cells were incubated at 4 °C with H18Ab (1:500) for 30 min binding, washed with serum-free medium, and transferred to 37 °C for 15 min uptake (to allow antigen uptake into early endosomes). After washing with cold PBS/1 M NaCI, the cells were transferred to 37 °C for a 30 min chase (to permit antigen trafficking into recycling compartments). Then, the cells were returned to the ice followed by washing with PBS/1 M NaCI (to remove the surface-recycled Ag-Ab complex) or not. After cells were fixed and permeabilized, the surface-recycled and intracellular non-recycled CD147-H18Ab complexes were stained with anti-mouse AF488 and confocal imaged as indicated above.

### Rab and Rac GTPase activation

GTPase activation was performed as previously described [9, 31]. Briefly, serum-starved cells were stimulated with HGF (20 ng/mL)-contained medium (0.5% FBS), lysed and incubated with GST-EEA1 (for Rab22 activation)- and GST-Rabaptin-5 (for Rab5 activation)-immobilized beads, respectively. After subjecting the collected pellets to SDS-PAGE, the GTP-bound Rab5 and Rab22 in samples were determined by Western blot. The Rac1-GTP level was assessed using GST-PAK-CRIB immobilized beads as previously described [29, 32, 33].

### Cell adhesion and aggregation

The processes were performed as previously described [8, 9, 34]. Cell adhesion: cells were detached with EDTA (0.02%), suspended in serum-free medium, added to Matrigel (5 mg/ml)-, collagen (10μg/mL)-, fibronectin (10μg/mL)- or laminin (10μg/mL)-coated 96-well plates (2 × 10^4^/well) and incubated for 30 min and 2 h, respectively. After removing the medium, attached cells were stained with 0.2% crystal violet, lysed with 5% SDS, and the absorbance was read at 540 nm. Slow aggregation assay: single-cell suspensions (2 × 10^5^/ml) seeded in agar-coated six-well plates were static incubated at 37 °C for 24 h. Photographs were taken under an inverted microscope. The degree of cell aggregation was scored as follows: solitary cells (≤2 cells), small and loose aggregates (3–20 cells), middle and compact aggregates (21–200 cells), and large compact aggregates (≥200 cells). In oder to block CD147 trafficking on the cell surface, anti-CD147 pcAb (1:1000) was added to Arf6(Q67L)-expressed cells.

### Transwell cell migration and invasion

Cell migration and invasion assays were performed using 24-well Transwell units with an 8-mm pore size polycarbonate filter (Millipore) according to previous method [9]. Briefly, 5 × 10^4^ cells were seeded into Matrigel-coated (5 mg/ml) or -uncoated culture inserts with medium containing 0.5% FBS. The lower chamber was filled with 0.5% FBS medium containing 20 ng/ml HGF as a chemoattractant. After 24 h incubation, cells remaining in the upper compartment were completely removed, whereas cells that invaded into the Matrigel and/or migrated out onto the lower surface of the membrane were fixed with 4% PFA and stained with 1% crystal violet. Ten fields were photographed for each group. Data were collected from three independent experiments, each performed in duplicate.

### Gelatin zymography

The process was performed as previously described [9]. The 24 h culture cell medium (serum-free) was 20 fold concentrated by using Amicon Ultra-4 10 k devices. Equivalent amounts of protein were separately loaded in SDS-PAGE gel containing 1% gelatin, washed by 2.5% Triton, activated by incubation buffer, and stained with 0.05% Coomassie blue.

### Western blot and immunofluorescence

The samples were quantified by the BCA kit, resolved by SDS-PAGE, blotted with prime Abs diluted as follows: anti-CD147 (H18, 1:2000), anti-Arf6 (1:500), anti-HA (1:1000), anti-ZO-1 (1:500), anti-E-cadherin (1:500), anti-pan-cadherin (1:500) and anti-β-catenin (1:500), anti-Rac1 (1:200), anti-Rab5 (1:500), anti-Rab22 (1:500), and anti-β-actin (1:1000). Immunofluorescent assays were performed as previously described [35], with Ab dilution as follows: anti-HA (1:200), anti-CD147 (H18, 1:200), anti-ZO-1 (1:100), and anti-E-cadherin (1:100).

### Patient samples and immunohistochemistry

Sixty HCC patient’s specimens were collected from the Third Central Hospital of Tianjin Medical University. All patients underwent successful hepatectomy and were not treated with radiotherapy or chemotherapy before operation. Overall survival was defined as the interval between surgery and death, or between surgery and the last observation point. Kaplan–Meier analysis was used for the survival data. Informed consents were obtained and this study was approved by the Medical Ethics Committee of the Hospital.

Immunohistological analysis was performed as previous reported with revision [36, 37]. Briefly, after hydrogen peroxide blocking, paraffin sections were microwave-heated for 15 min in Tris-EDTA buffer (10 mM Tris-HCI, 1 mM EDTA, pH 9.0), blocked with goat serum, and incubated with mouse anti-CD147 Ab (1:100), anti-Arf6 Ab (1:50), rabbit anti-Rac1 Ab(1:30), and anti-ARNO Ab (1:30) overnight at 4 °C. Non-immune mouse or rabbit IgG was used as the negative control. Immunohistochemistry was performed with the Envision™ two step system (Dako, USA). Sections were treated with 3, 3-diaminobenzidine and counterstained with haematoxylin. Immunopositivity was independently evaluated by two pathologists, who were blinded to the clinical data, and scored as follows: low staining (negligible, or 1+ positivity regardless of positive cell percentages, or 2+ positivity of < 30% of cells), high staining (2+ positivity of ≥30% of cells, or 3+ positivity of ≤50% of cells, or 3+ positivity of > 50% of cells).

### Bioinformatics and statistical analysis

Microarray mining analysis as performed based on the GEPIA (Gene Expression Profiling Interactive Analysis) database (http://gepia.cancer-pku.cn/) [38]. The ANOVA differential method was used for tumor (T) vs paired normal (N) samples. Confocal microscopy, flow cytometry, and western blot data were derived from three independent experiments. All data were analyzed using GraphPad Prism 5 software and are described as the mean ± SD values.

## Results

### Arf6 up-regulates the endocytic recycling of CD147 by activating Rab5 and Rab22

**Fig. 1:**
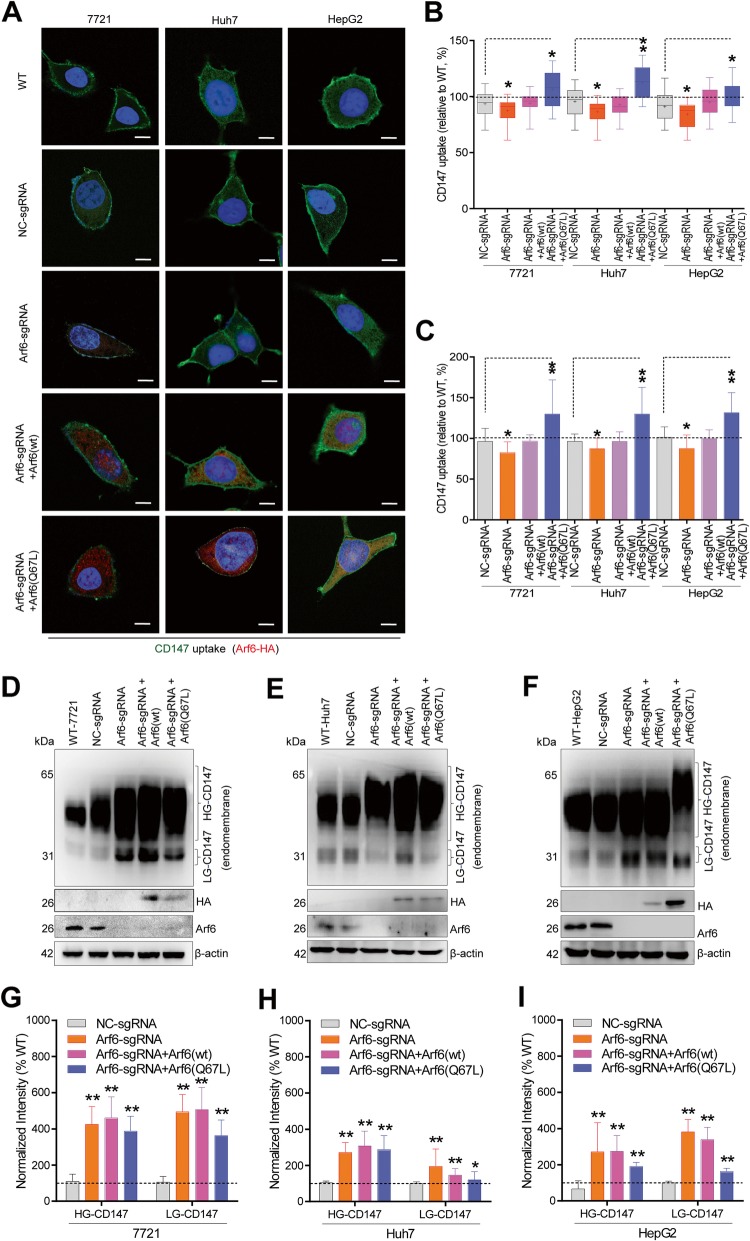
Arf6-KD accumulated CD147 in the endomembrane compartments. Arf6-perturbed liver cancer cells were incubated with the H18Ab-AF488 complex at 37 °C for 15 min uptake, then quickly rinsed with PBS/1 M NaCl, fixed, permeabilized, stained by anti-HA Ab, and visualized under a confocal microscope. **a** Representative observations are shown. Scale bar: 20 um. **b** Box-and-whiskers plots depict the uptake of the H18Ab-AF488 complex in cell populations. The CD147 uptake in untreated cells (WT) was set as 100%. Significant differences compared with NC-KD cells are shown. **c** Uptake of the H18Ab-AF488 complex in Arf6-perturbed cells was quantified by flow cytometry. *n* = 3. HG-, and LG-CD147 were blotted from the endomembrane fractions of Arf6-perturbed liver cancer cells. Representative results from three independent experiments are shown (**d**, **e**, **f**), and corresponding quantitative scans of CD147 blots were analyzed (**g**, **h**, **i**). The CD147 level in untreated cells (WT) was set as 100%. Significant differences compared with NC-KD cells are shown. *n* = 3. * *P* < 0.05, ** *P* < 0.01

We made three kinds of shRNA-lentivirus for human Arf6 gene silencing, and tested their silencing efficiency in commonly used liver cancer cell lines. Treatment of cells with the A1 lentivirus long-term knocked-down Arf6 expression to levels < 5% of those in control cells (Additional file 1: Figure S1). Therefore, we used this A1-lentivirus throughout this study. Under this condition of treatment, cell viability was not affected (data not shown). However, CD147 uptake was slightly reduced by Arf6-KD (Fig. 1a, b). Extraneous over-expression of Arf6(wt) and Arf6(Q67L) both reversed the uptake reduction of CD147 in Arf6-KD cells. This regulatory action of Arf6 on CD147 uptake was also confirmed by flow cytometry results (Fig. 1c). As Arf6(Q67L) over-expression excessively reversed CD147 uptake, we explored this phenomenon by checking the endomembrane-associated CD147 level. Unexpectedly, Western blot results showed that Arf6-KD markedly increased endomembrane-resident CD147 levels. In addition, further over-expression of Arf6(wt) or Arf6(Q67L) did not diminish CD147 accumulating in the endomembrane of Arf6-KD cells (Fig. 1d-i). These results suggested that Arf6 intervention can affect CD147 uptake and its subsequent trafficking process in liver cancer cells.

**Fig. 2:**
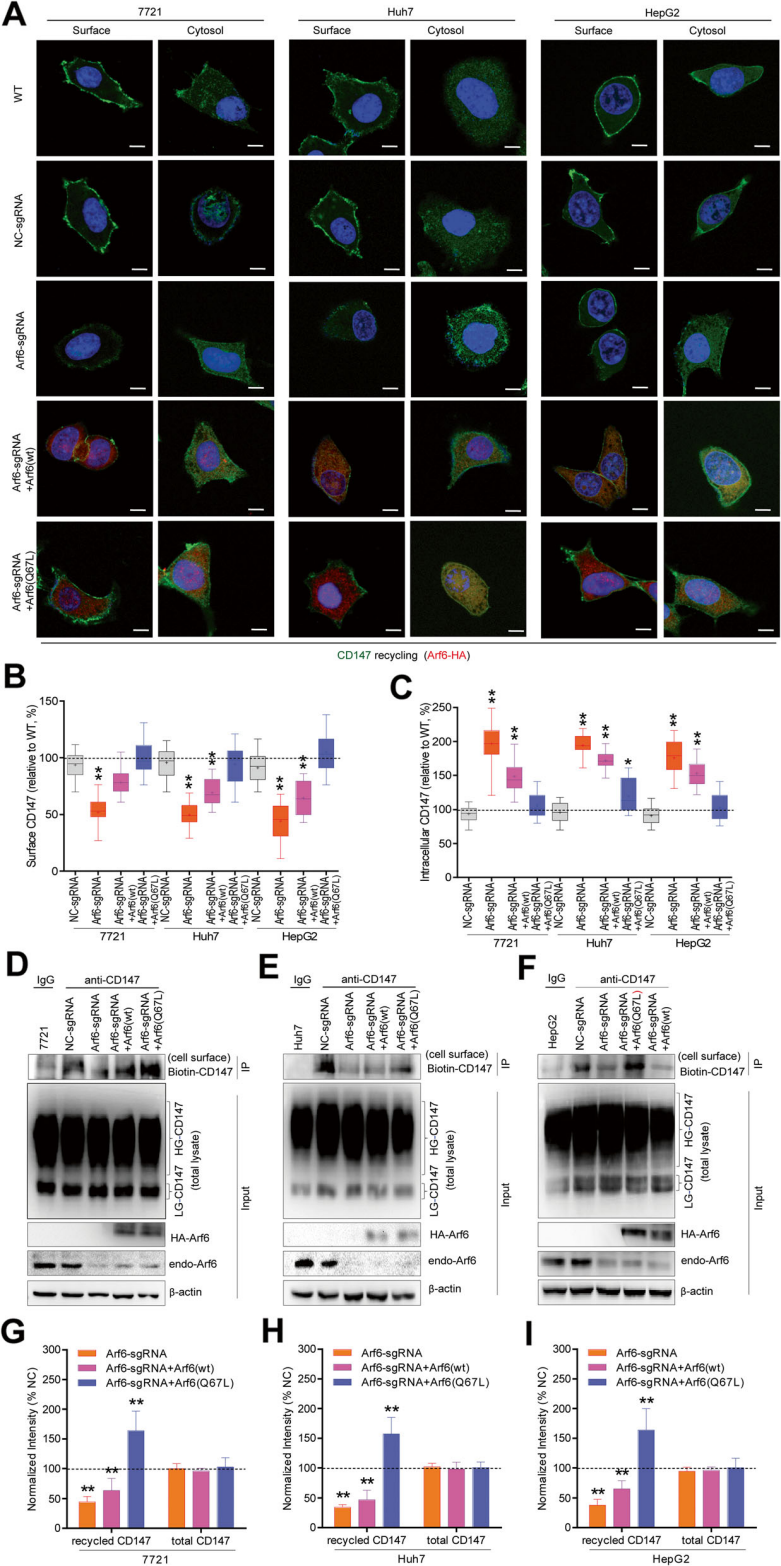
Arf6-KD decreased CD147 recycling to the cell surface. Arf6-perturbed liver cancer cells were incubated with H18Ab for uptake at 37 °C. After washing surface-residual Ab with PBS/1 M NaCl, cells were chased for 30 min and the recycled CD147-H18Ab complex on the cell surface was stained with anti-mouse AF488 and imaged under a confocal microscope. In parallel, chased cells were further washed with PBS/1 M NaCl to remove the surface-recycled portion, then fixed, permeabilized, and the intracellular non-recycled complex (cytosol) was stained and confocal imaged. **a** Representative observations are shown. Scale bar: 20 um. **b**, **c** Box-and-whiskers plots depict the surface (recycled) and cytosol (non-recycled) CD147 pool in cell populations. The recycled or non-recycled CD147 pool in untreated cells (WT) was set as 100%. Significant differences compared with NC-KD cells are shown. Western blot determined the biontin-CD147 level after cell surface biotinylation, avidin binding, and DTT elution. Representative blot results from three independent experiments are shown (**d**, **e**, **f**), and corresponding quantitative scans of CD147 blots were analyzed (**g**, **h**, **i**). The cell-surface biotin-CD147 level in untreated cells (WT) was set as 100%. Significant differences compared with NC-KD cells are shown. *n* = 3. ** *P* < 0.01

**Fig. 3:**
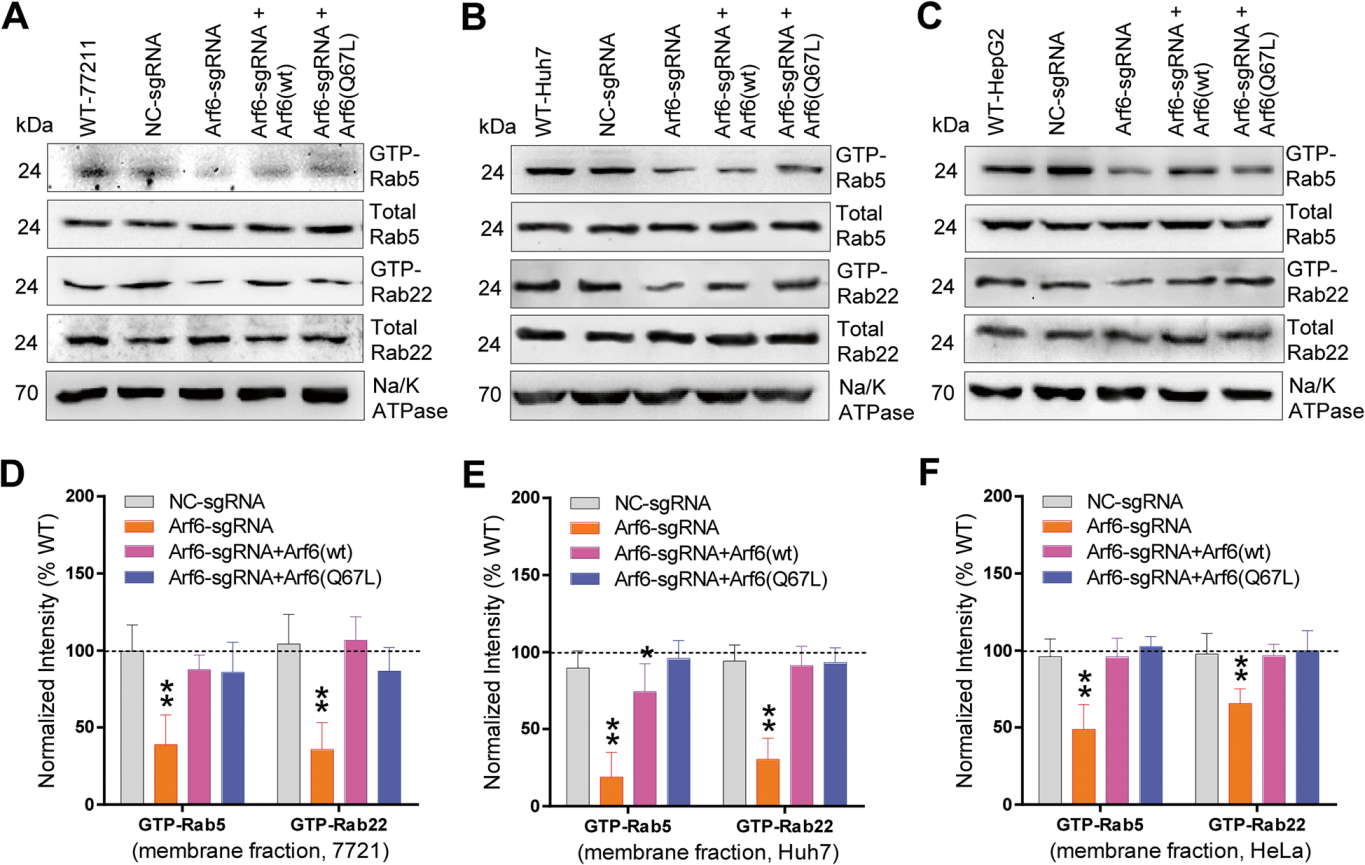
Arf6-KD impaired the activation of Rab5 and Rab22. Arf6-perturbed liver cancer cells were serum-starved, and stimulated with HGF-contained medium, lysed immediately to extract membrane fractions, and incubated with GST-EEA and -Rabenosyn-5 beads, respectively. Activated Rab GTPases in pull-down precipitates were blotted with corresponding Abs. Representative blot results from three independent experiments are shown (**a**, **b**, **c**). Activated Rab was quantified and corresponding data were analyzed (**d**, **e**, **f**). The activated Rab level in untreated cells (WT) was set as 100%. Significant differences compared with NC-KD cells are shown. n = 3. * *P* < 0.05, ** *P* < 0.01

Intracellular accumulation of membrane protein has been widely supposed to inhibit exocytosis, and therefore the effect of Arf6 on CD147 recycling was investigated. Confocal imaging and flow cytometry results showed that Arf6-KD significantly decreased the recycled pool of CD147 on the cell surface, and simultaneously increased the cytoplasmic non-recycled CD147 pool (Fig. 2a-c, Additional file 1: Figure S2). Conversely, further over-expression of Arf6(Q67L) completely restored CD147 recycling in liver cancer cells. In contrast, over-expression of Arf6(wt) partially relieved Arf6-KD-reduced CD147 recycling. Notably, Western blot checking of the CD147 level in surface-biotinylated cells showed that Arf6-KD markedly impaired CD147 recycling to the cell surface, and this impairment could be reversed by Arf6(Q67L) over-expression (Fig. 2d-i). As endomembrane-associated Rab5 and Rab22 GTPases dominate the CD147 endocytic recycling [9], we then checked whether Arf6 intervention affected their activation. Pull-down results showed that Arf6-KD significantly attenuated Rab5 and Rab22 activation in the membranous fraction, and such attenuations were rescued by Arf6(wt) or Arf6(Q67L) over-expression (Fig. 3). Together, these data suggested that Arf6 expression promotes the endocytic recycling of CD147 in liver cancer cells, which largely occurs through concurrent activation of Rab5 and Rab22.

### Arf6-mediated CD147 recycling promotes cell adhesion, aggregation, and junction formation

**Fig. 4:**
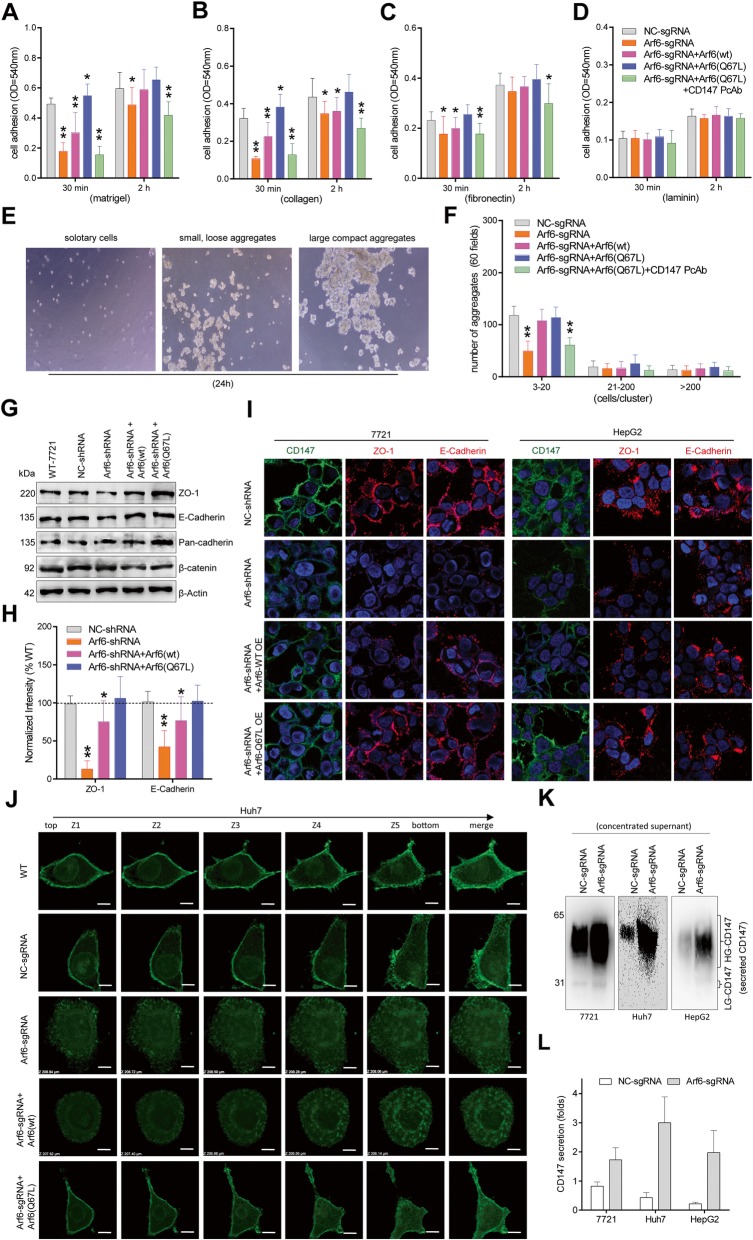
Arf6-mediated CD147 recycling facilitates cell adhesion, aggregation, and tight junction formation. Arf6-perturbed 7721 cells were detached and reseeded on Matrigel- (**a**), collagen- (**b**), fibronectin- (**c**) or laminin- (**d**) coated plates, and the attached cell number was determined. Three independent experiments were performed in quintuplicate. Significant differences compared with NC-KD cells are shown. OD: optical density. Arf6-perturbed 7721 cells were reseeded on agar substrate for 24 h static culture. Cell aggregation clusters were evaluated microscopically. **e** Representative light microscopy pictures are shown (×40). **f** The number of different-sized clusters was compared among groups. Sixty fields from three independent experiments were counted and analyzed. Significant differences compared with NC-KD cells are shown. Arf6-perturbed 7721 cells were confluent grown. Expressions of tight junction marker (ZO-1) and adherent junction marker (E-cadherin) were determined. Representative blot results from three independent experiments are shown (**g**). Protein bands were quantified, and the expression level in untreated cells (WT) was set as 100%. Significant differences compared with NC-KD cells are shown (**h**). * *P* < 0.05, ** *P* < 0.01. In parallel, the treated cells were respectively stained with H18Ab (coupled with anti-mouse AF488), ZO-1 Ab (coupled with anti-rabbit AF647), and E-cadherin Ab (coupled with anti-mouse AF647), and viewed by confocal microscopy. Optical sections at the plane of cell junctions are shown (**i**). Vertical (x/y) fields of CD147-stained Huh7 cells along the z-axis are also shown (**j**). Supernatants of cultured cells were concentrated (20 fold) and loaded for CD147 blotting. Representative results from three independent experiments are shown (**k**), and corresponding quantitative data were analyzed (**l**)

Rapid recycled CD147, following its endocytosis, promotes the adhesion capability of cell [4]. To determine the contribution of Arf6-mediated CD147 recycling on this aspect, the ECM (extracellular matrix) attachment of Arf6-perturbed cells was examined. Arf6-KD significantly reduced the number of 7721 cells attached to Matrigel, collagen, and fibronectin, but not to laminin (Fig. 4a-d). Overexpression of Arf6(wt) or Arf6(Q67L) rescued these decrements to varying degrees. Notably, when Arf6-KD 7721 cells were pre-blocked with anti-CD147 pcAb, the rescue effect induced by Arf6(Q67L) was relieved (Fig. 4a-c). Such Arf6-intervention-induced adhesion changes to Matrigel, collagen and fibronectin were also observed in Huh7 and HepG2 cells (Additional file 1: Figure S3). As CD147 concentrated on the cell surface causes cell self-aggregation [6], we then checked whether Arf6-mediated CD147 recycling contributed to cell-cell adhesion. Arf6-KD significantly weakened the formation of small-sized cell aggregates but did not affect the establishment of middle- and large-sized cell aggregates (Fig. 4e, f and Additional file 1: Figure S4). This weakening effect was rescued by Arf6(wt) or Arf6(Q67L) over-expression, and pre-blocking the cells with anti-CD147 pcAb again relieved the rescue effect. These data suggested that Arf6-mediated CD147 recycling is essential to liver cancer cell-matrix and cell-cell adhesion.

To check whether Arf6-mediated CD147 recycling affected intercellular junctions, expression of key junction proteins were investigated. Western blot results showed that Arf6-KD significantly suppressed the levels of the tight junction marker ZO-1 and the adheren junction marker E-cadherin (Fig. 4g, h). Notably, extraneous over-expression of Arf6(wt), especially Arf6(Q67L), rescued the Arf6-KD-reducted ZO-1 and E-cadherin levels. In contrast, levels of pan-cadherin and β-catenin were not affected by Arf6 intervention. Correspondingly, ZO-1 staining markedly disappeared due to Arf6-KD-induced CD147 diminishing on the cell surface (Fig. 4i). Attenuated E-cadherin staining was also observed in Arf6-KD cells but not detected in those further overexpressed with Arf6(wt) or Arf6(Q67L). Seeing that CD147 is generally targeted to the basolateral membrane in epithelial cells [39], we next checked the apical-basal turnover of CD147. In Huh7 cells, CD147 was polarized to the basolateral membrane, and was translocated to the apical membrane when Arf6 was depleted (Fig. 4j). Interestingly, over-expression of Arf6(Q67L) but not Arf6(wt) restored the targeting of CD147 to the basolateral membrane. In addition, the apical recycling of CD147 in Arf6-KD cells correlated with an enhanced CD147 secretion level (Fig. 4k, l). These data revealed the tight-link between Arf6-mediated CD147 recycling and liver cancer cell adhesion, aggregation and junction stability.

### Arf6-mediated CD147 recycling facilitates liver cancer cell migration and invasion

**Fig. 5:**
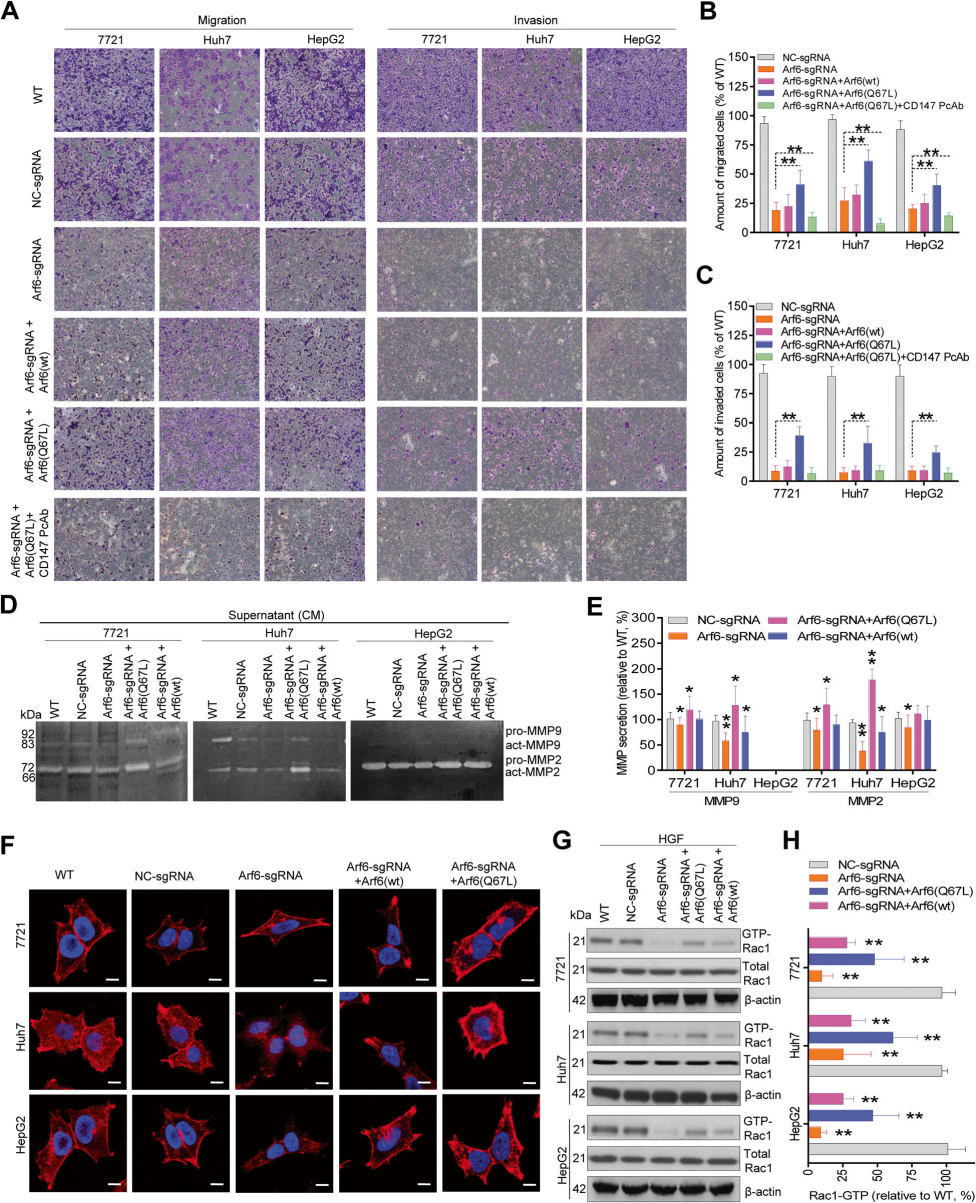
Arf6-mediated CD147 recycling promotes cell migration and invasion. **a** Transwell migration and invasion analysis. Equal numbers of Arf6-perturbed liver cancer cells were seeded into the upper chamber coated without or with Matrigel. Twenty-four hours later, cells migrating or invading through the Transwell filters were crystal-violet-stained and counted under a microscope. Representative results from three independent experiments are shown. The number of migrated or invaded cells in untreated groups (WT) was set as 100% for comparison. **d** MMPs activity analysis. Secreted MMPs in the concentrated medium (CM) were measured by gelatin zymography. Representative results from three independent experiments are shown. The MMPs secreted from untreated groups (WT) was set as 100% for comparison. **f** Cytoskeleton reorganization analysis. Arf6-perturbed cells were reseeded in poly-D-lysine-coated chamber slides, serum-free starved, fixed, permeabilized, and stained with Rhodamine-phalloidin. Representative confocal images from three experiments are shown. Scale bar: 20 um. **g** Rac1 activation analysis. Arf6-perturbed cells were serum-starved, lysed, and incubated with CRIB beads. The activated Rac1 in pull-down precipitates were immunoblotted, and representative results from three independent experiments are shown. The Rac1 activation level in untreated groups (WT) was set as 100% for comparison. **b**, **c**, **e**, **h** Cell counts and protein bands were quantified by Image J software, and significant differences compared with NC-KD cells are shown: n = 3, ** *P* < 0.01, * *P* < 0.05

Impaired cell adhesion and junction formation are important steps during cancer progression [35]. To directly assess the role of Arf6-mediated CD147 recycling, the migratory and invasive behaviors of liver cancer cells were examined. Arf6-KD yielded a significant reduction in cell migration and invasion capacity in all three liver cancer cell lines. Unexpectedly, these two characteristics were partially restored by further over-expression of Arf6(Q67L) but not Arf6(wt) (Fig. 5a-c). When Arf6-KD cells were blocked with anti-CD147 pcAb, the Arf6(Q67L)-induced rescue effect disappeared, suggesting CD147 recycled to the cell-surface dictates the migration and invasion behaviors of liver cancer cells. Since CD147-triggered ECM-degradation is a prerequisite for liver cancer invasion, MMPs secretion from Arf6-perturbed cells was examined. Gelatin zymography results showed that Arf6-KD significantly decreased MMP2 and MMP9 secretion, and such decrements were rescued by Arf6(Q67L) over-expression (Fig. 5d, e). Because cytoskeleton reorganization and membrane ruffling are required for cell migration, we further checked the cell morphological change after Arf6 intervention. As expected, Arf6-KD markedly decreased the formation of polymerized actin stress fiber and lamellipodia numbers in liver cancer cells. Interestingly, the resulting cortical thin actin microfilament bundles were only reversed by Arf6(Q67L) expression. In contrast, further over-expression of Arf6(wt) and Arf6(Q67L) re-established lamellipodia formation (Fig. 5f and Additional file 1: Figure S5). Since Rac1 is a downstream target of Arf6 and responsible for lamellipodia formation, we further checked the membrane-associated Rac1 activity in Arf6-perturbed cells, and Arf6-dependent Rac1 activation stimulated by HGF was detected (Fig. 5g, h). These results strongly suggested that Arf6-mediated CD147 recycling is required for liver cancer cell migration and invasion.

### Arf6-CD147 signaling correlates with a poor clinical outcome of liver cancer patients

**Fig. 6:**
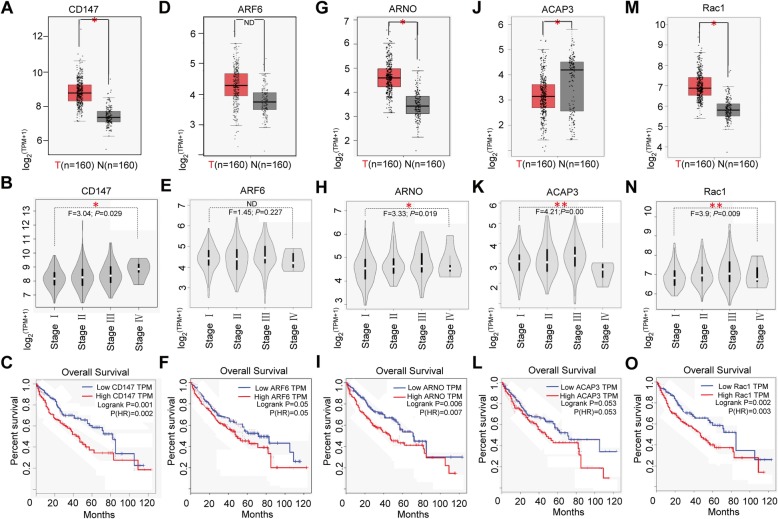
Significance of Arf6-mediated CD147 signaling components expressed in liver cancer. The mRNA expression level of CD147, ARF6, ARNO, ACAP3, and Rac1 in clinical samples was determined by GEPIA. **a**, **d**, **g**, **j**, **m** Box plots depict the expression difference between liver cancer (T) and normal tissues (N). Matched TCGA normal & GTEx data are included. Each dot represents the expression level of a sample. **b**, **e**, **h**, **k**, **n** Stage plots depict the expression variation among different pathological stages of liver cancer tissues. **c**, **f**, **i**, **l**, **o** Survival plots depict the overall survival (OS) for low- and high-expression groups of the signal genes among liver cancer patients (cutoff: median). log-rank test, *p < 0.05, ** *P* < 0.01

To investigate the clinical significance of Arf6-mediated CD147 recycling, we determined the expression level of their signaling components in liver cancer patients. GEPIA bioinformatics analysis showed that CD147, Rac1 and an Arf6-specific GEF ARNO exhibited higher transcription levels in liver cancer tissues than in matched normal liver tissues (Fig. 6a, g, m), whereas, the mRNA level of Arf6 and other Arf6-specific GEFs or GAPs (except ACAP3) tended to be no different (Fig. 6d and Additional file 1: Figure S6). Further checking their mRNA level in different pathological stages showed that CD147, ARNO, and Rac1 expressions were markedly up-regulated, while ACAP3 expression was significantly down-regulated at stage IV of liver cancer (Fig. 6b, h, k, n). Kaplan-Meier survival curves showed that higher expressions of CD147, ARNO, and Rac1 mRNA were significantly associated with shorter overall survival (OS), whereas Arf6 mRNA expression was slightly associated with the survival of liver cancer patients (Fig. 6c, f, i, o). Further co-expression network analysis showed that CD147 highly expressed in liver cancer tissues correlates with Arf6, ARNO, and Rac1 expressions. Interestingly, certain endosome trafficking-associated genes (e.g. ATP6V1F, SNX6, and SOS2) and protein quality control genes (NEMF, MAP 4 K5, and ZNF410) were co-enriched with the Arf6-mediated CD147 functional connection (Additional file 1: Figure S7). These data suggested that complex trafficking machinery is involved in Arf6-mediated CD147 recycling, and the Arf6-CD147 signaling components could be survival predictors for liver cancer patients.

**Fig. 7:**
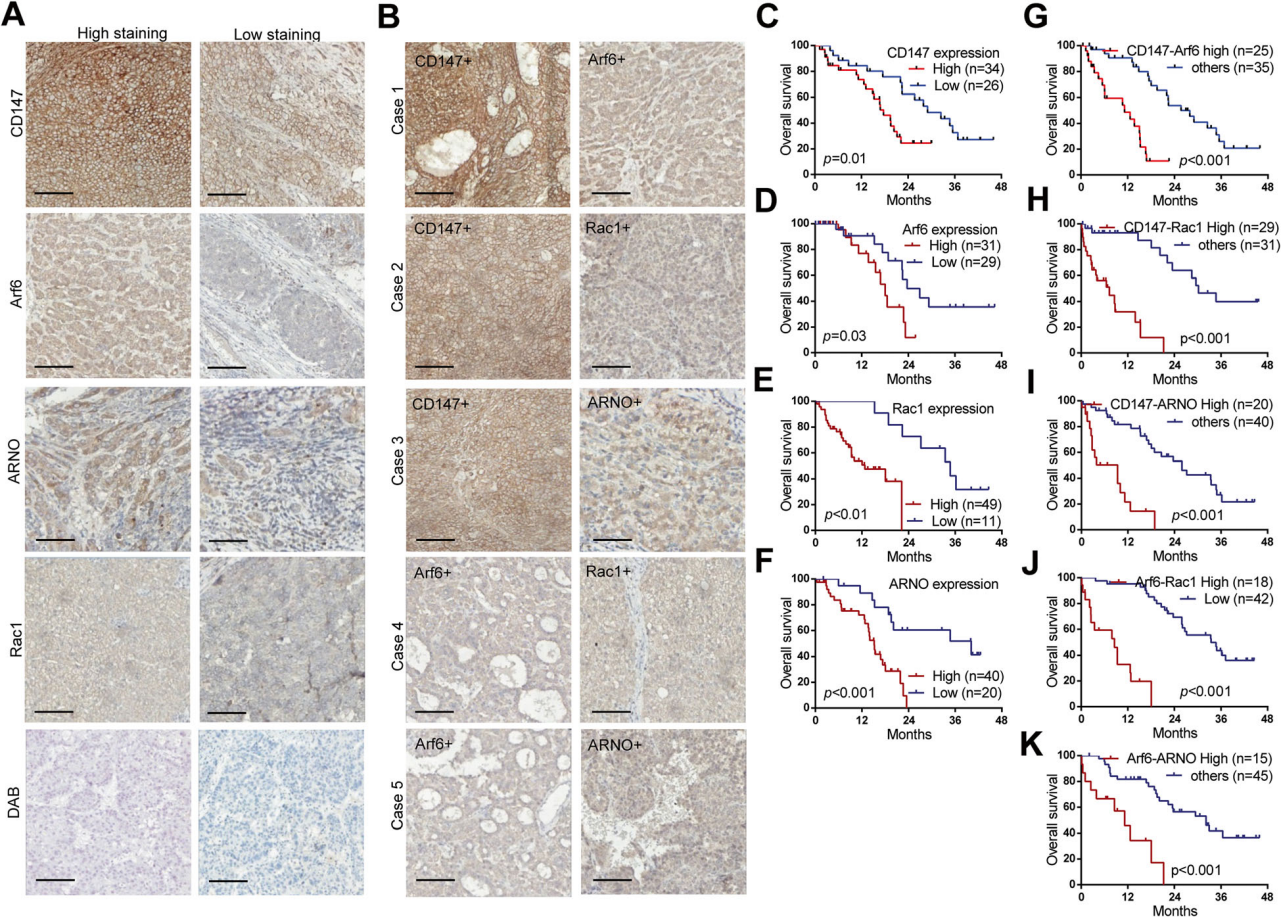
High-expression of Arf6-CD147 signaling components correlates with poor outcome of HCC patients. **a** Representative images of varied immunohistochemical staining (high- vs low-) of CD147, Arf6, ARNO and Rac1 in human HCC specimens. Scale bars, 200um. **b** Double positive staining of CD147-Arf6 (Case 1), CD147-Rac1 (Case 2), CD147-ARNO (Case 3), Arf6-Rac1 (Case 4) and Arf6-ARNO (Case 5) in well differentiated HCC tissue. Kaplan-Meier curves of the overall survival of HCC patients, with regard to the expression levels of each single component of Arf6-CD147 signal pathway (**c**-**f**), and with regard to the simultaneous high-expression of two components (**g**-**k**), as indicated. *P* values represent the results of the log-rank test

To further verify the clinical relevance for the above results, we collected surgical specimens from HCC patients, and investigated the expression profile of Arf6-CD147 signaling components. Immunohistochemical results showed that the expression (that is, staining) levels of CD147, Arf6, ARNO, and Rac1 varied among specimens of primary HCCs, each from a different patient. Intra-tumor heterogeneity may exist in HCC, which seemed to be reflected by the non-uniform expression of the Arf6 and ARNO, even within the same cancerous lesion (Fig. 7a). On the other hand, CD147 and Rac1 appeared to be expressed rather uniformly within each lesion and among different lesions (Fig. 7a, b). The clinicopathological parameters of HCC patients at the time of hepatectomy are summarized in Table. 1 and Additional file 1: Table S1. By classifying the specimens based on the expression level of the Arf6-CD147 signaling components, we found that advanced TNM stages were significantly associated with high expressions of CD147, ARNO, and Rac1. High expressions of CD147 and Arf6 were positively associated with portal vein tumor thrombus, and a high level of serum AFP was associated with Arf6 and ARNO high expressions (Table. 1). Simultaneous high expression of two of these signaling components exhibited higher association with these three features (Additional file 1: Table S1). Notably, high expression of each of these proteins tightly correlated with poor overall survival of HCC patients (Fig. 7c-f), in which simultaneous high expression of two of these proteins exhibited a higher correlation (Fig. 7g-k and Additional file 1: Figure S8). Therefore, our results suggested that high expression of the signaling components of the Arf6-CD147 pathway provides excellent biomarkers predictive of poor outcomes of HCC patients.

**Table 1:** Clinicopathological features of HCC patients and association with Arf6-CD147 signaling components

Feature	CD147-high % (n)	Arf6-high % (n)	ARNO-high % (n)	Rac1-high % (n)
All patients (*n* = 60)	57 (34)	52 (31)	67 (40)	82 (49)
Gender				
Male (*n* = 45)	56 (25)	53 (24)	67 (30)	82 (37)
Female (*n* = 15)	60 (9)	47 (7)	67 (10)	80 (12)
*P*	0.76	0.66	1.00	0.85
Age at surgery (years)				
<59 (*n* = 34)	65 (22)	50 (17)	62 (21)	85 (29)
>59 (*n* = 26)	46 (12)	54 (14)	73 (19)	77 (20)
*P*	0.15	0.77	0.36	0.41
TNM stage				
1, 2 (*n* = 22)	36 (8)	36 (8)	45(10)	55 (12)
3, 4 (*n* = 38)	68 (26)	61 (23)	79 (30)	97 (37)
*P*	0.02	0.07	0.01	< 0.01
Portal vein tumor thrombus				
+(*n* = 6)	100 (6)	100 (6)	100 (6)	100 (6)
- (*n* = 54)	52 (28)	46 (25)	63 (34)	80 (43)
*P*	0.02	0.01	0.07	0.22
Histological grade				
G1,2 (*n* = 53)	55 (29)	49 (26)	64 (34)	85 (45)
G3,4 (*n* = 7)	71 *(5)*	71 (5)	86 (6)	57 (4)
*P*	0.40	0.27	0.26	0.07
AFP (ng/mL)				
<400 (*n* = 46)	57 (26)	39 (18)	59 (27)	87 (40)
≥400 (*n* = 14)	57 (8)	93 (13)	93 (13)	64 (9)
*P*	0.97	< 0.01	0.02	0.05
Maximal tumor size (cm)				
<5 (*n* = 42)	55 (23)	45 (19)	69 (29)	83 (35)
≥5 (*n* = 18)	61 (11)	67 (12)	61 (11)	78 (14)
*P*	0.65	0.13	0.55	0.61
Background liver status				
With cirrhosis (*n* = 40)	60 (24)	58 (23)	70 (28)	75 (30)
Without cirrhosis (*n* = 20)	50 (10)	40 (8)	60 (12)	95 (19)
*P*	0.46	0.20	0.44	0.06
Capsule formation				
+(*n* = 35)	57 (20)	43 (15)	57 (20)	77 (27)
- (*n* = 25)	56 (14)	64 (16)	80 (20)	88 (22)
*P*	0.93	0.11	0.06	0.28

*P* values represent the results of the Chi-square test

## Discussion

Compared with much research on Arf6-mediated clathrin-dependent trafficking [2, 19, 20, 22], Arf6-driven clathrin-independent trafficking events have been less studied. Previous studies using HeLa cell as the model reported that Arf6 does not contribute to the uptake of the CIE cargo, but its inactivation is required for cargo sorting soon after entry and Arf6 activation is essential for the recycling of the CIE cargo [2]. CD147 is a typical ‘A-cargo’ protein that uses CIE to enter cells and directly recycles to the cell surface [9, 15]. Here, we found that Arf6 intervention slightly influenced CD147 uptake but markedly affected its recycling (Fig. 1a-c, Fig. 2a-c and Additional file 1: Figure S2), which resulted in CD147 being highly present on the surface of liver cancer cells. Further over-expression of the Arf6(Q67L) active-mutant completely reversed Arf6-KD-reduced CD147 endocytic recycling, highlighting that Arf6 activation can facilitate both the endocytosis and the recycling of CD147. Similar to the observation in HeLa cells [2, 18, 40], CD147 was accumulated in the endomembrane when Arf6 was depleted or further overexpression of Arf6(wt) or Arf6(Q67L) (Fig. 1d-f). This Arf6 mutant-induced endosome-trapping mirrors with its excessive reversion effect on CD147 uptake, strongly suggesting that cyclic activation and inactivation of Arf6 are required for the endocytic recycling of CD147.

Intracellular trafficking of ‘A-cargo’ CIE proteins is regulated by certain Rab GTPases [2, 18]. Generally, Rab5 activation boosts early steps of CD147 uptake, and Rab22 activation accelerates the direct recycling of CD147 to the cell surface [24, 25]. We found that Arf6-KD reduced Rab5 and Rab22 activation in liver cancer cells, and such reductions were recovered by Arf6(wt), especially Arf6(Q67L) over-expression (Fig. 3). To our knowledge, this is the first report on Arf6 expression acting on Rab activation. As Rab22 is responsible for sorting ‘A-cargo’ proteins away from the Rab5-associated endosomes and into tubular recycling endosomes [18, 41], the phenomenon that Arf6-KD reduced CD147 recycling is logical. On the other hand, because Rab5 is the central endosome Rab defining initial sorting events [41], Arf6(Q67L)-induced Rab5 over-activation that leads to CD147 trapped in the CIE endosomes is a legitimate inference.

Recycled endosomes return membrane proteins back to the cell surface which is important for cell adhesion [22]. Previous studies revealed the contribution of CD147 to cell adhesion with a direct knock-down or over-expression strategy [42–46]. We found that Arf6-mediated CD147 recycling promotes liver cancer cells adhering to key ECM-components (Fig. 4a-c, and Additional file 1: Figure S3). CD147 decrease on the cell surface reduced cell adhesion to collagen and fibronectin but not to laminin, suggesting that the endocytic recycling of ECM-bound molecules (including but not limited to CD147) are differentially regulated by Arf6. In epithelial cells, Arf6 is an important regulator of intercellular adhesion and CD147 plays a significant role in adhesion modulation of liver cancer cells [47, 48]. It was found that Arf6-KD reduced CD147 recycling which impaired the cell-cell contact and prevented the aggregation of liver cancer cells (Fig. 4e, f and Additional file 1: Figure S4). Reduced cell-matrix adhesion and weakened cell-cell contact facilitate liver cancer cells acquiring the migration phenotype (Fig. 5).

To initiate local tissue invasion, epithelial cancer cells have to detach from the primary site by disassembling the cell-cell junction. Using polarized epithelial cells as the model, most studies showed that Arf6 activation promotes E-cadherin uptake from cell-cell contact sites to early endosomes, which leads to the disassembly of adherent junctions [20, 22, 47]. Here, we observed E-cadherin being significantly diminished or degraded in Arf6-KD liver cancer cells. In contrast, with further over-expression of Arf6(wt) or Arf6(Q67L), a recovered E-cadherin level was detected (Fig. 4g). This phenomenon implies that adequate activation of Arf6 is critical for stimulating the turnover of E-cadherin. ZO-1 is a key tight junction protein involved in the establishment of hepatic cell polarity [49]. Although a study reported that CD147 depletion results in a ZO-1 increase in prostate cells [42], we found that Arf6-KD reduced CD147 recycling diminished ZO-1 in liver cancer cells, and Arf6 activation restored ZO-1 turnover (Fig. 4g-i). As Rab22 and Rac1 are activated by Arf6 (Fig. 3, Fig. 5g), and they direct the traffic of junction proteins to cell-cell contact sites via mutual signal crosstalk [21], it is most likely that Arf6 intervention changed the selective targeting of CD147 and the retention of ZO-1 and E-cadherin to lateral membranes (Fig. 4g-j), which cooperatively triggered the disassembly of functional junctions between adjacent liver cancer cells.

**Fig. 8:**
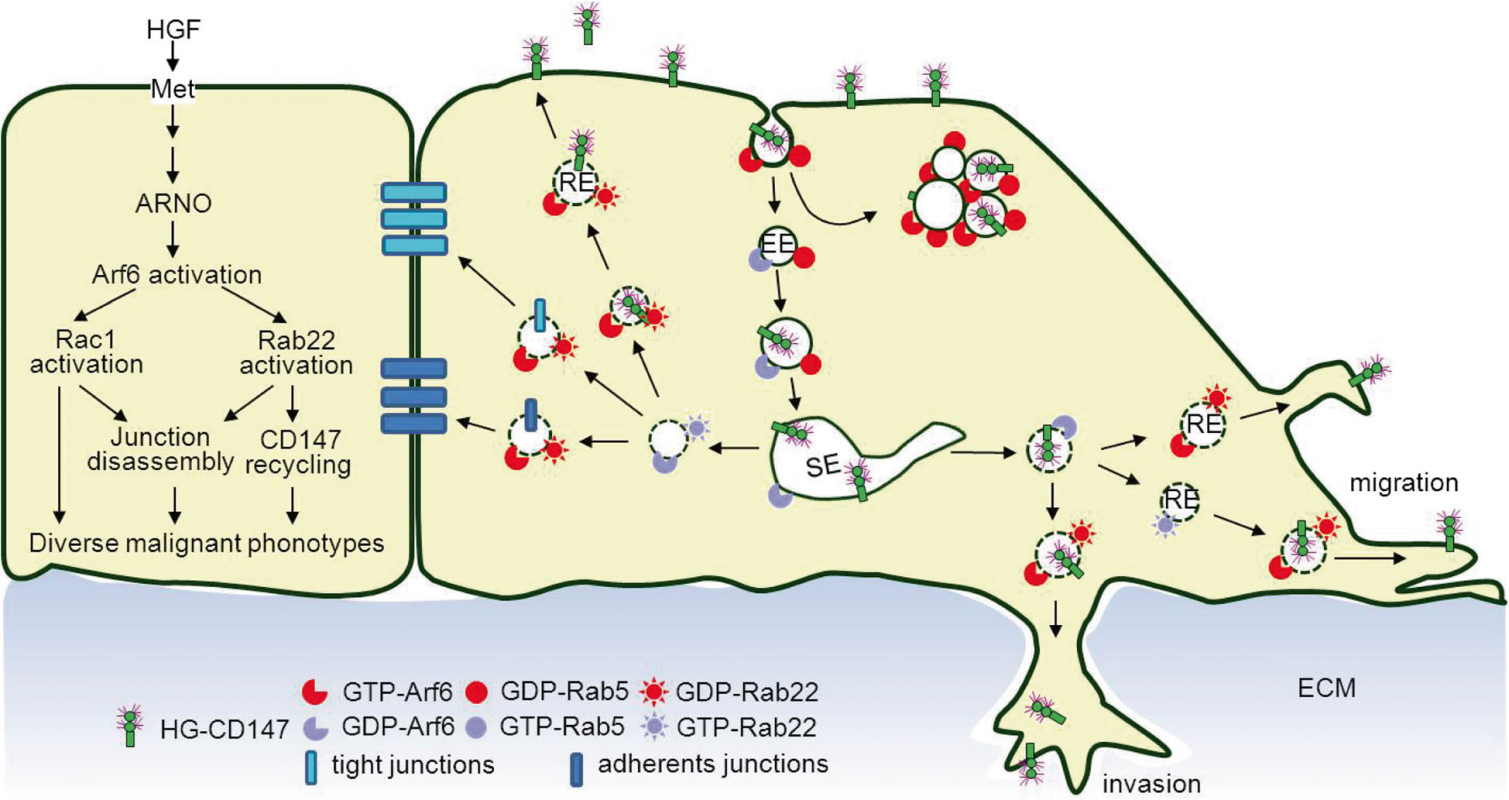
Working model for HCC migration and invasion controlled by Arf6-driven endocytic recycle of CD147. Arf6 is activated in response to HGF, which is produced in and secreted from liver cancer cells, and promotes CD147 turnover by endocytic recycling. Arf6 activation induces CD147 to highly present at the cell surface and markedly destabilize the junctions at cell-cell contacts, which promote liver cancer cells spreading, migrating, and invading into the ECM. The Arf6 GEF, ARNO, probably activates Arf6 on endosomes, which triggers Rab22 and Rac1 activation (other effectors may also be activated), and subsequently facilitate multiple steps of CD147 trafficking

Rac1 has been described as a downstream target of Arf6, whereas the Arf6-dependent Rac1 regulation seems to be complex and varies upon cell status. In non-stimulated HEK-293 and MDCK cells, Arf6 depletion leads to Rac1 activation [50, 51], suggesting Arf6 is responsible for maintaining Rac1 in an inactive state before cell scattering. However, we did not detect such a trend in liver cancer cells (data not shown). This might be because the Rac1-GEFs were not efficiently recruited to the target membrane when cells were absent of stimulation with the pro-metastatic factor. Actually, in HGF-stimulated liver cancer cells, we found that Arf6-KD inhibited Rac1 activation and further over-expression of Arf6(Q67L) partially restored such inhibition (Fig. 5g, h). These results are similar to previous findings in EGF-stimulated breast cancer cells, serum-stimulated glioma cells, and HGF-stimulated MDCK cells [52–54]. Seeing that ARNO is the only Arf6-specific GEF highly expressed in liver cancer tissues (Figs. 6, 7), it is most probably that HGF activates Arf6 by recruiting ARNO to the endomembrane (including endosomes), which subsequently triggers the activation of Rac1 and other downstream effectors (e.g. phospholipase D) through an as yet unknown mechanism. The Rac1 activation at membrane compartments may induce multiple events, including changes in cell morphology, formation of actin-based lamellipodia, separation from neighboring cells, and finally exhibit a dramatic increase in migration and invasion of liver cancer cells (Fig. 8).

Although CD147 and Arf6 significant expression in liver cancer were already reported [5, 7, 8, 10, 48, 55, 56], we found for the first time that expression of the CD147-assocaited Arf6-ARNO-Rac1 signal axis in liver cancer tissues was significantly higher than in surrounding non-tumorous tissues (Fig. 6a-m, and Fig. 7a, b). Markedly, high expression of Arf6-CD147 signaling components was significantly correlated with more aggressive characters, in terms of advanced TNM stage, portal vein tumor thrombus, high AFP level, and short overall survival, which are putative clinicopathological markers for HCC development, invasiveness, and unfavorable prognosis (Fig. 7c-k, Table. 1, and Additional file 1: Table S1). These data strongly revealed that active Arf6-CD147 signaling occurs in HCC and is tightly associated with the HCC malignant phenotype and renders it as a potential excellent biomarker. Further studies are required to gain insight into the details of this signaling, and to develop new therapies targeting this Arf6-driven CD147 trafficking pathway.

## Conclusions

In summary, our study demonstrates that Arf6 is essential for the endocytic recycling of CD147 and its mediated malignant phenotypes in liver cancer cells. Moreover, we provide evidence supporting that high expression of the Arf6-CD147 signaling components are tightly correlated with poor overall survival of HCC patients.

## Supplementary information

**Additional file 1: Figure S1.** CD147 expression and stable knock-down in liver cancer cells. **Figure S2.** Flow cytometry analysis of CD147 level on liver cancer cell surface. **Figure S3.** Arf6-mediated CD147 recycling promotes Huh7 and HepG2 cell adhesion to ECM. **Figure S4.** Arf6-KD impaired the cell-cell aggregation of Huh7 and HepG2 cells. Arf6-perturbed cells were reseeded on agar for static culture, and cell aggregation clusters were evaluated. **Figure S5.** Morphometric analyses of Arf6-perturbed liver cancer cells. **Figure S6.** ARF6-specific GEFs and GAPs expressed in liver cancer patients. Box plots depict the expression level difference between liver cancer (T) and normal tissues (N). **Figure S7.** Co-expression network analysis of the Arf6-CD147 gene pair. **Figure S8.** Pair-wise correlation analysis for the expression (IHC staining) levels of CD147, Arf6, Rac1 and ARNO in primary HCC tissues. **Table S1.** Clinicopathological features of HCC patients and association with co-expression of CD147, Arf6, ARNO, and Rac1.

## Data Availability

Please contact the corresponding author for all data requests.

## Abbreviations

Ab: antibody
Arf6: ADP-ribosylation factor 6
CIE: clathrin-independent endocytosis
CME: clathrin-mediated endocytosis
ECM: extracellular matrix
GAP: GTPase-activating protein
GEF: guanine nucleotide exchange factor
H18Ab: anti-CD147 monoclonal antibody 18
HCC: hepatocellular carcinoma
KD: knock-down
MMPs: matrix metalloproteases
