# Supplementary material for: Arf6-driven endocytic recycling of CD147 determines HCC malignant phenotypes
Source: J Exp Clin Cancer Res. 2019 Nov 21;38:471. doi: 10.1186/s13046-019-1464-9 (PMC6868876; doi:10.1186/s13046-019-1464-9)
Supplement: Supplementary file 1 — Additional file 1: Figure S1. CD147 expression and stable knock-down in liver cancer cells. Figure S2. Flow cytometry analysis of CD147 level on liver cancer cell surface. Figure S3. Arf6-mediated CD147 recycling promotes Huh7 and HepG2 cell adhesion to ECM. Figure S4. Arf6-KD impaired the cell-cell aggregation of Huh7 and HepG2 cells. Arf6-perturbed cells were reseeded on agar for static culture, and cell aggregation clusters were evaluated. Figure S5. Morphometric analyses of Arf6-perturbed liver cancer cells. Figure S6. ARF6-specific GEFs and GAPs expressed in liver cancer patients. Box plots depict the expression level difference between liver cancer (T) and normal tissues (N). Figure S7. Co-expression network analysis of the Arf6-CD147 gene pair. Figure S8. Pair-wise correlation analysis for the expression (IHC staining) levels of CD147, Arf6, Rac1 and ARNO in primary HCC tissues. Table S1. Clinicopathological features of HCC patients and association with co-expression of CD147, Arf6, ARNO, and Rac1. [file 13046_2019_1464_MOESM1_ESM.docx]

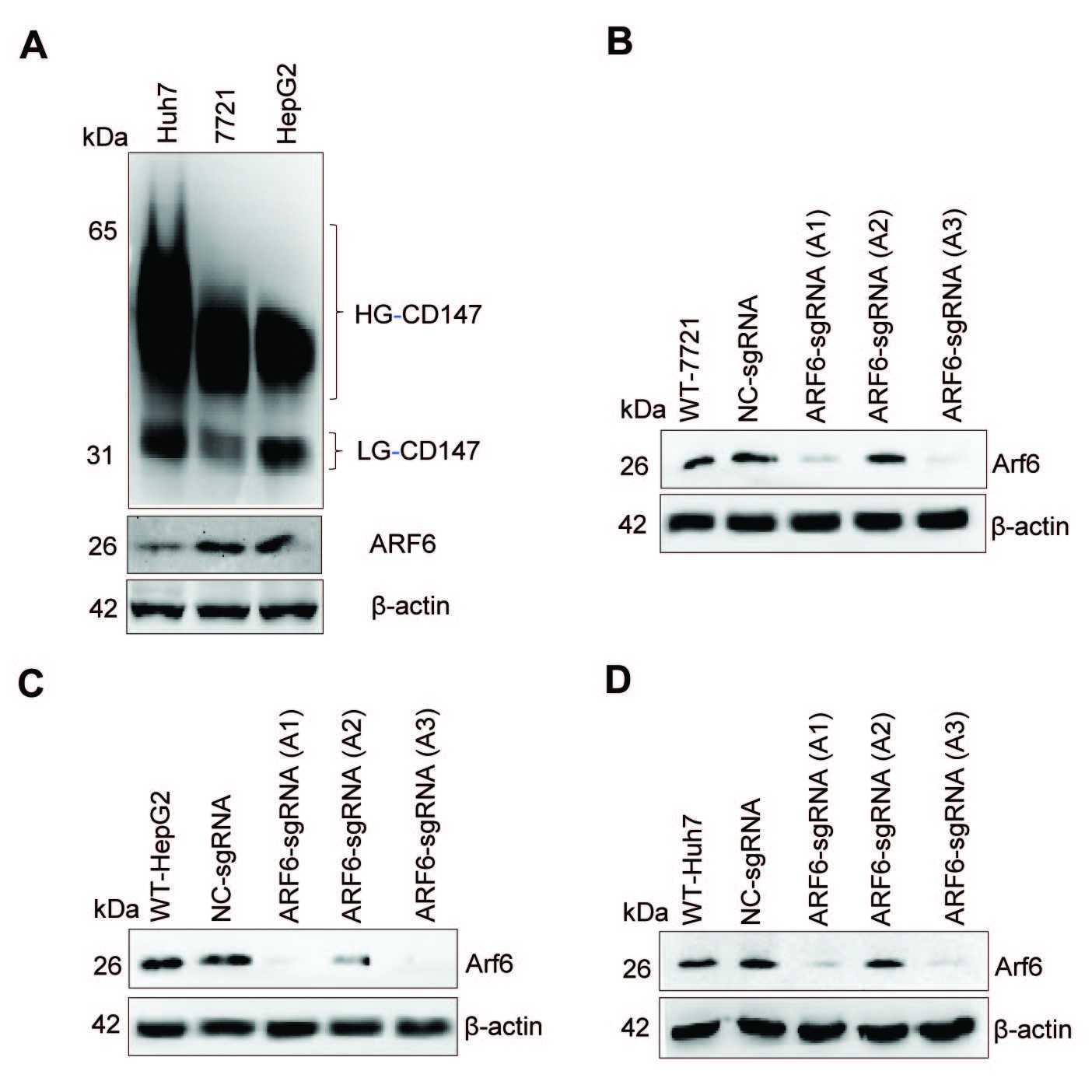


**Fig. S1** CD147 expression and stable knock-down in liver cancer cells. (**A**) Western blot determined CD147 and Arf6 expressed in indicated liver cancer cells. (**B-D**) Cells were transfected with designed Arf6-shRNA lentivirus, and the stable knock-down effect was checked by Western blot. Representative results from three independent experiments are shown.


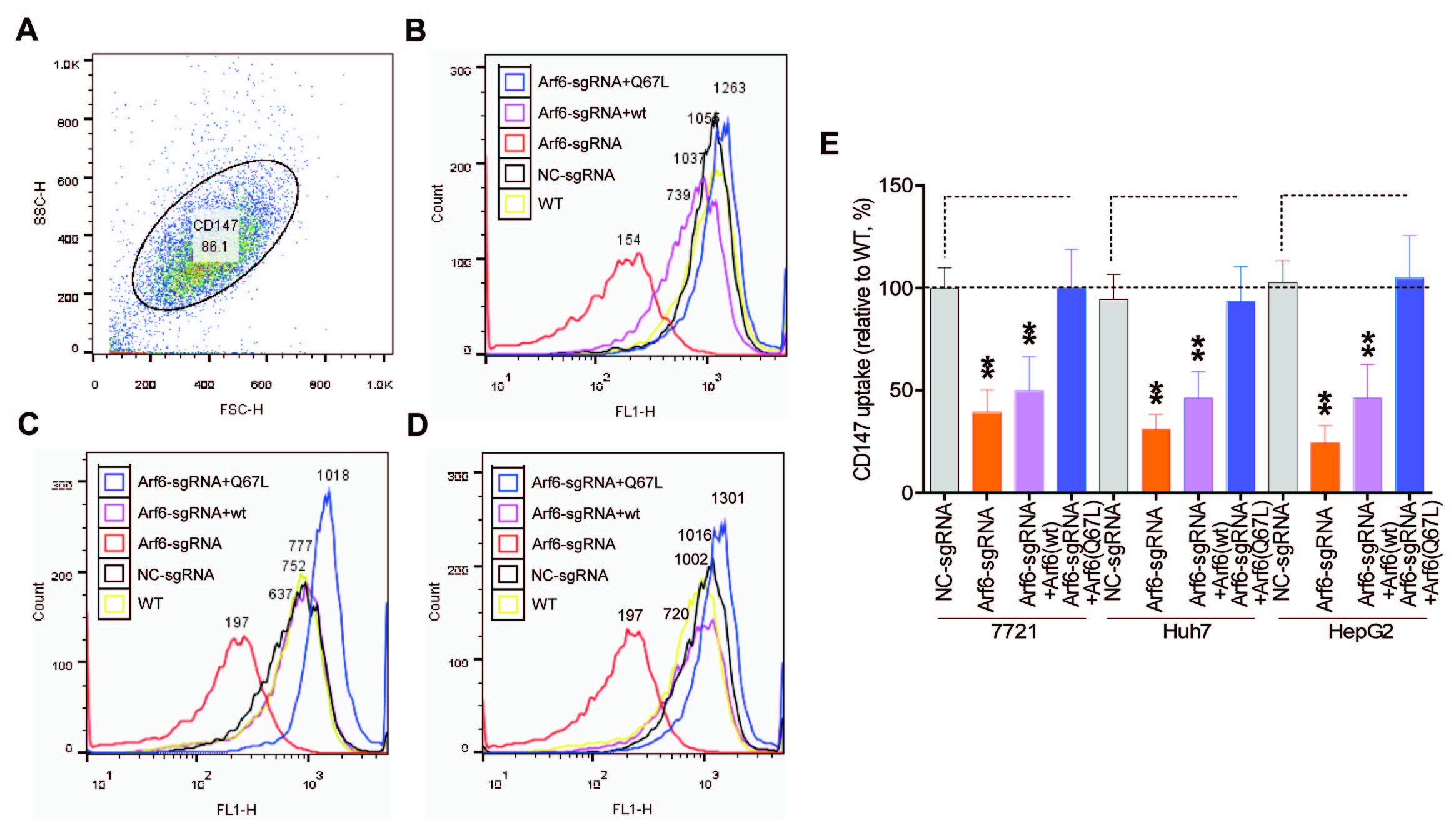


**Fig. S2** Flow cytometry analysis of CD147 level on liver cancer cell surface. Representative dot plot (**A**) and overlay histograms of 7721 (**B**), Huh7 (**C**) and HepG2 (**D**) cells are shown. The binding of H18Ab-AF488 to Arf6-perturbed liver cancer cells was quantified (**E**). n=3. ** *P* <0.01.


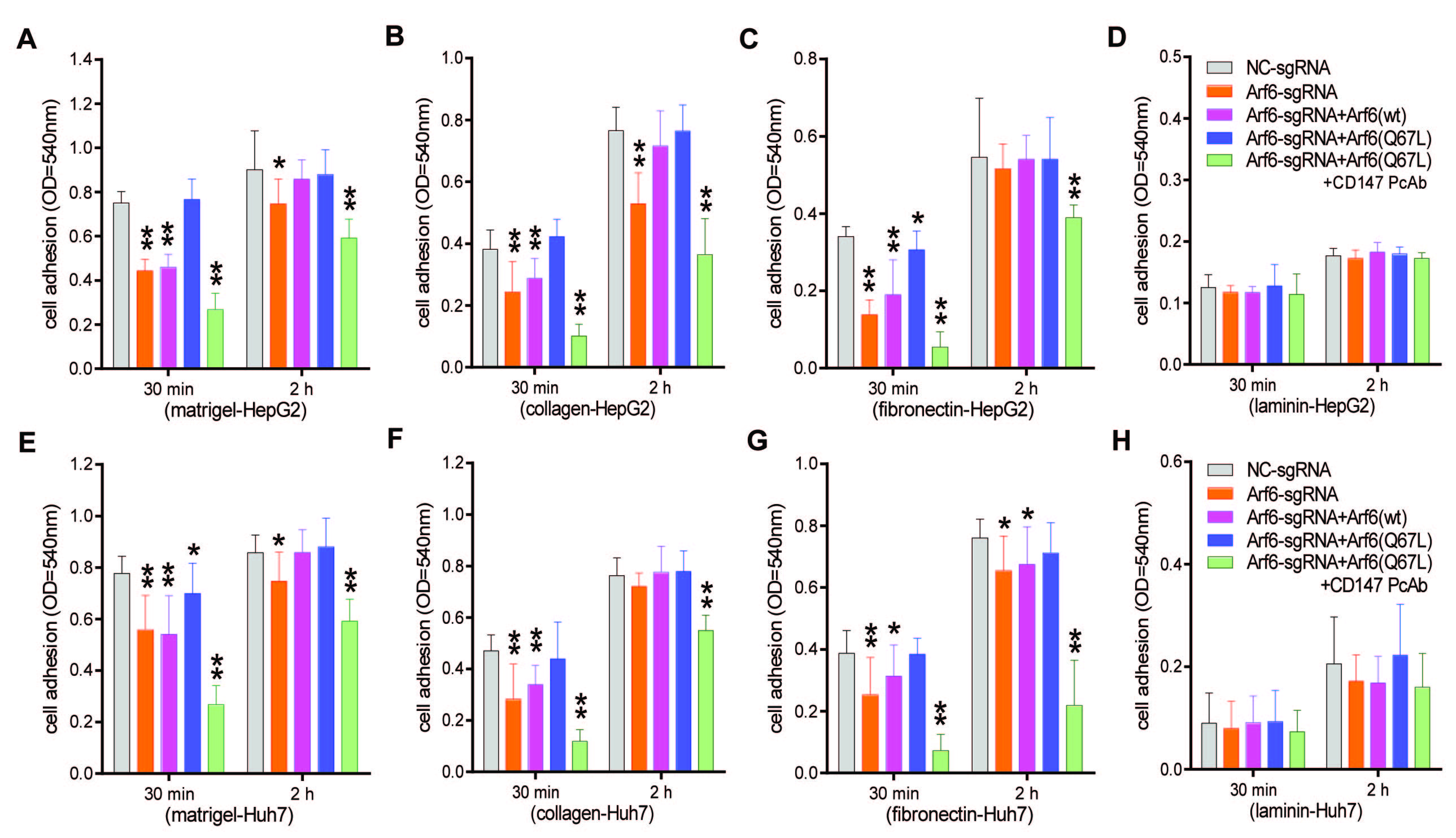


**Fig. S3** Arf6-mediated CD147 recycling promotes Huh7 and HepG2 cell adhesion to ECM. Arf6-perturbed cells were reseeded on Matrigel (**A**, **E**), collagen (**B**, **F**), fibronectin (**C**, **G**), or laminin (**D**, **H**)-coated plates for culture. The attachment of cells was determined. Significant differences compared with NC-KD cells are shown. n=3. * *P* <0.05, ** *P* <0.01.


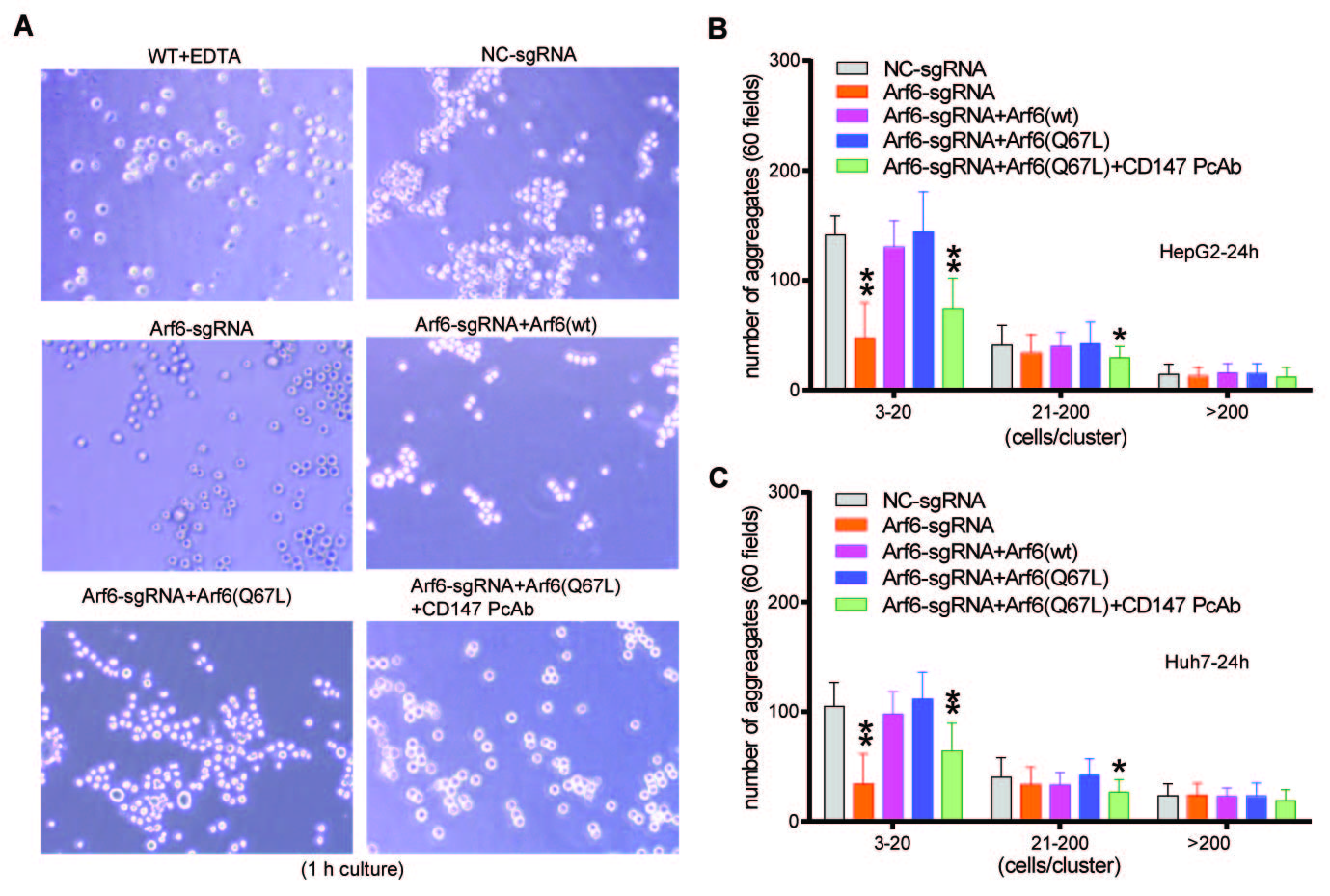


**Fig. S4** Arf6-KD impaired the cell-cell aggregation of Huh7 and HepG2 cells. Arf6-perturbed cells were reseeded on agar for static culture, and cell aggregation clusters were evaluated. (**A**) Representative microscope pictures of HepG2 cells after 1h static culture. (**B**) Number of cell clusters (24h culture) was compared among groups. Significant differences compared with NC-KD cells are shown. n=3. * *P* <0.05, ** *P* <0.01.


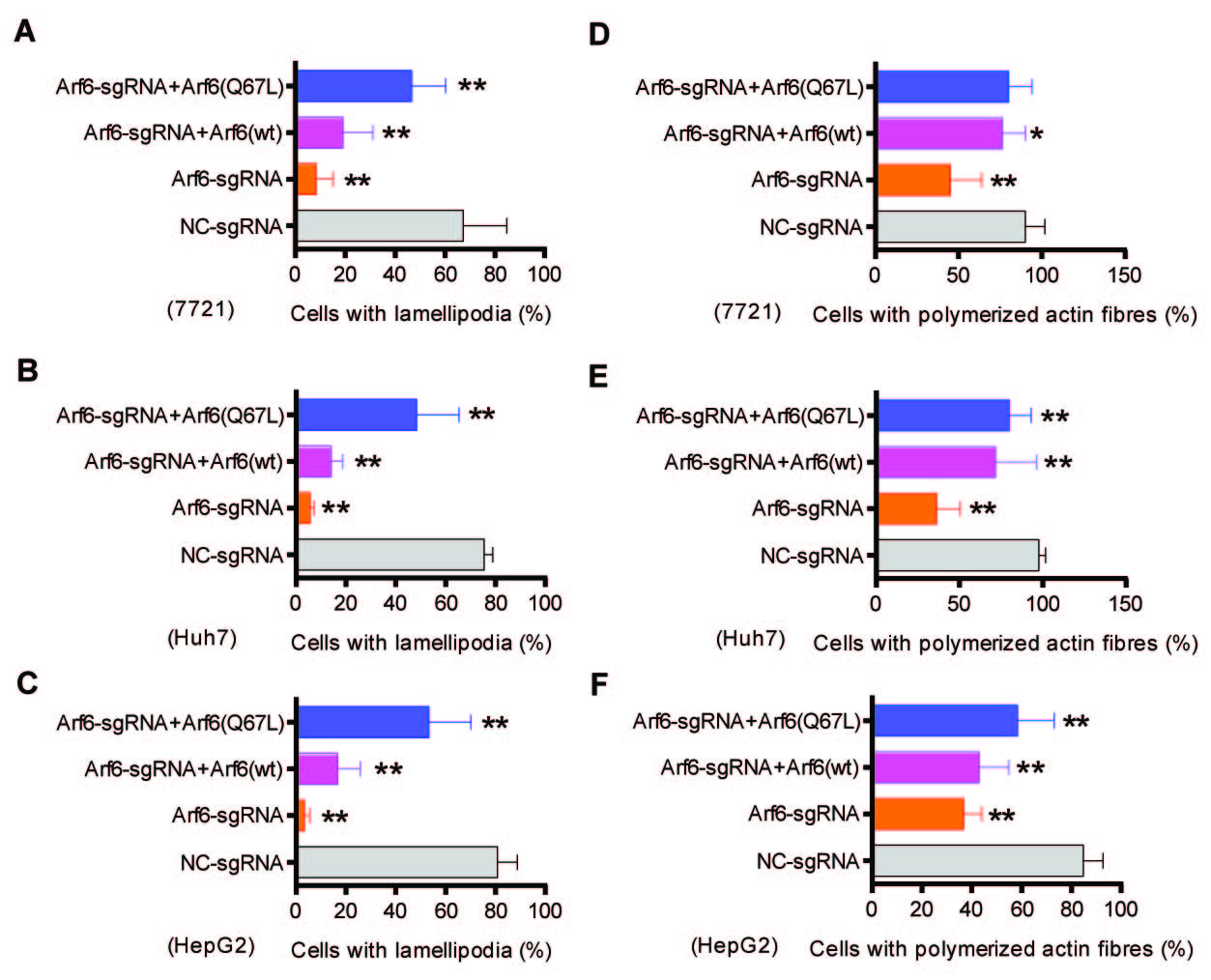


**Fig. S5** Morphometric analyses of Arf6-perturbed liver cancer cells. The number of cells with lamellipodia and polymerized stress fiber in untreated groups (WT) was set as 100%. 80 cells were counted for each group. Significant differences compared with NC-KD cells are shown: n=3, ** *P* <0.01, * *P* <0.05.


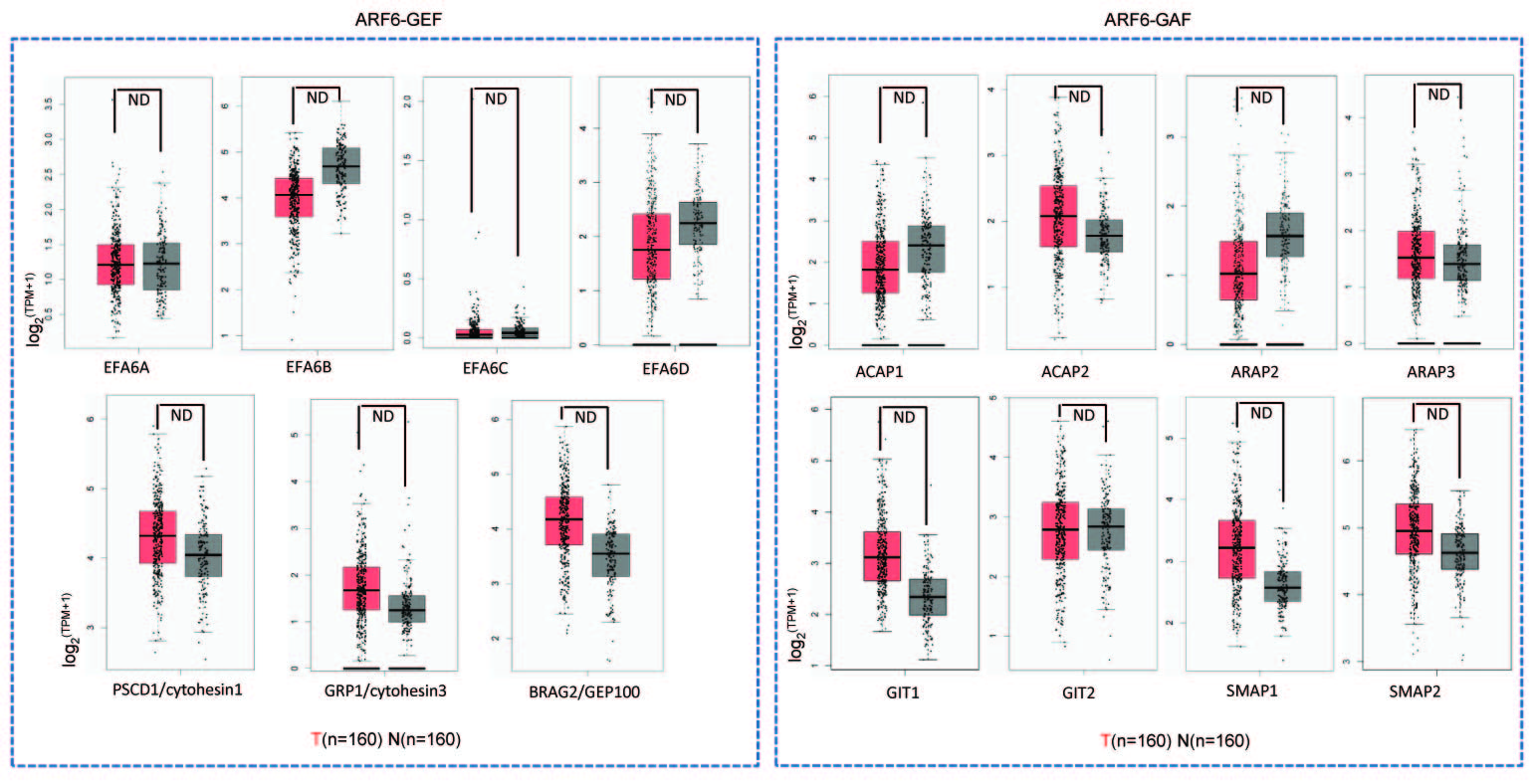


**Fig. S6** ARF6-specific GEFs and GAPs expressed in liver cancer patients. Box plots depict the expression level difference between liver cancer (T) and normal tissues (N). Matched TCGA normal & GTEx data are included. ND: no significant difference.


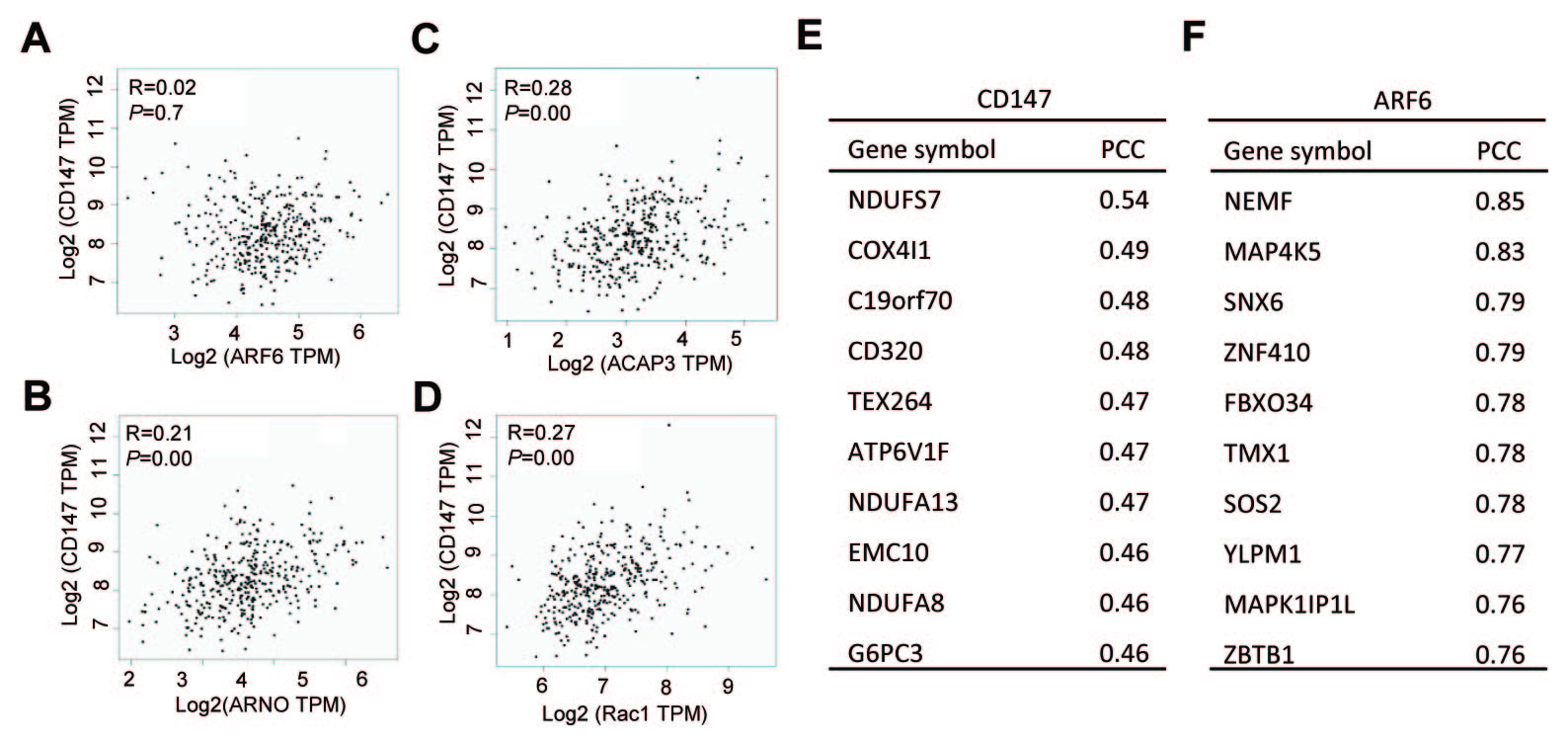


**Fig. S7** Co-expression network analysis of the Arf6-CD147 gene pair. (**A**, **B**, **C**, **D**) Pair-wise gene expression correlation analysis based on the TCGA & GTEx data. Pearson’s r test. (**E**, **F**) Function-associated gene detection based on the datasets mentioned above. The lists show the top ten genes with the most relevant function.


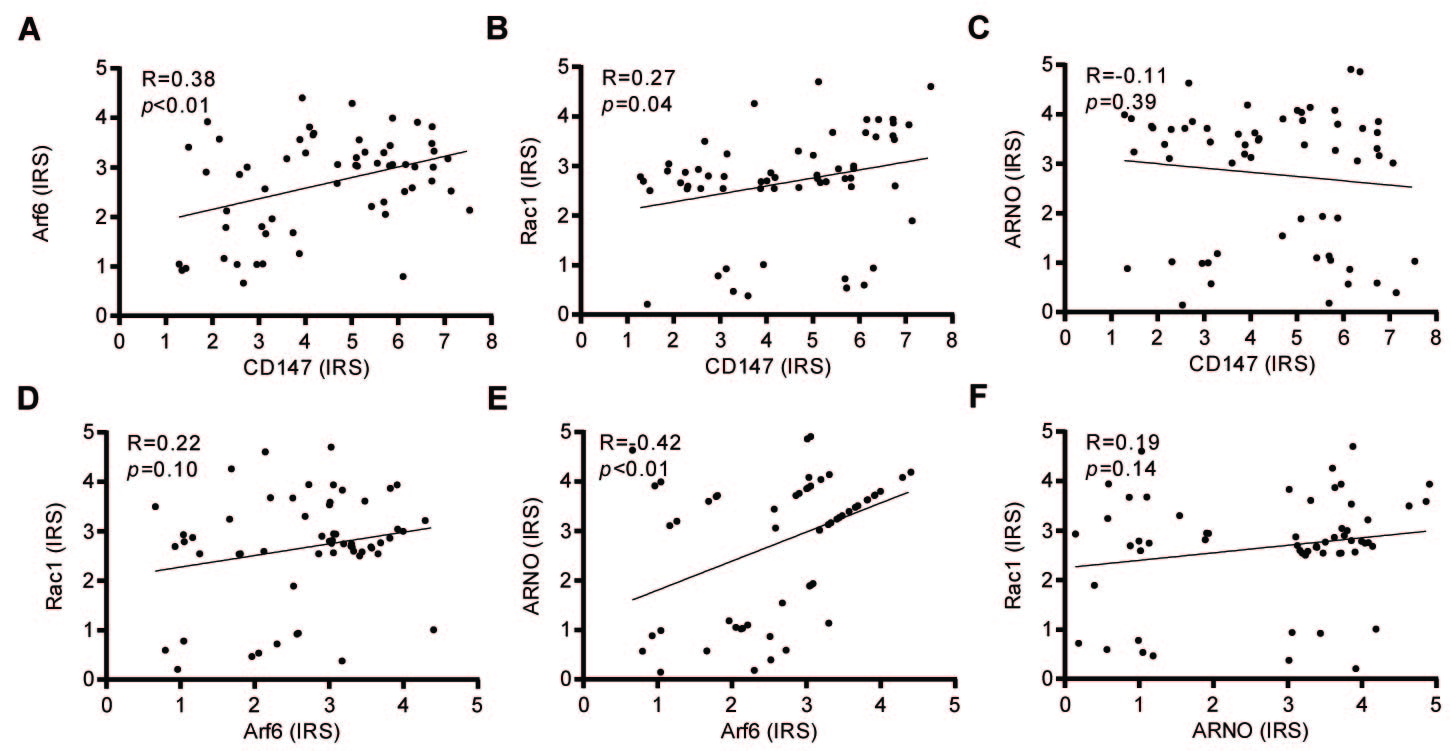


**Fig.S8** Pair-wise correlation analysis for the expression (IHC staining) levels of CD147, Arf6, Rac1 and ARNO in primary HCC tissues. The immunoreactive score (IRS) was calculated as the intensity of the staining reaction multiplied by the percentage of positive cells. Pearson’s r test.

**Table S1** Clinicopathological features of HCC patients and association with co-expression of CD147, Arf6, ARNO, and Rac1.

| Feature | CD147+Arf6 high  % (n) | CD147+Rac1 high  % (n) | CD147+ARNO high  % (n) | Arf6+Rac1 high  % (n) | Arf6+ARNO high  % (n) |
| --- | --- | --- | --- | --- | --- |
| All patients (n=60) | 42 (25) | 48 (29) | 33 (20) | 30 (18) | 25 (15) |
| Gender  Male (n=45)  Female (n=15)  *P* | 42 (19)  40 (6)  0.88 | 44 (20)  60 (9)  0.29 | 38 (17)  20 (3)  0.21 | 31 (14)  27 (4)  0.75 | 22 (19)  40 (6)  0.88 |
| Age at surgery (years)  <59 (n=34)  >59 (n=26)  *P* | 41 (14)  42 (11)  0.93 | 56 (19)  38 (10)  0.18 | 32 (11)  35 (9)  0.85 | 29 (10)  31 (8)  0.91 | 26 (9)  23 (6)  0.76 |
| TNM stage  1, 2 (n=32)  3, 4 (n=28)  *P* | 25 (8)  61 (17)  < 0.01 | 25 (8)  75 (21)  < 0.01 | 19 (6)  50 (14)  0.01 | 19 (6)  43 (12)  0.04 | 9 (3)  43 (12)  < 0.01 |
| Portal vein tumor thrombus  + (n=6)  - (n=54)  *P* | 100 (6)  35 (19)  < 0.01 | 100 (6)  43 (23)  < 0.01 | 100 (6)  26 (14)  < 0.01 | 100 (6)  22 (12)  < 0.01 | 100 (6)  17(9)  < 0.01 |
| Histological grade  G1,2 (n=53)  G3,4 (n=7)  *P* | 42 (22)  43 (3)  0.95 | 47 (25)  57 (4)  0.62 | 32 (17)  43 (3)  0.57 | 26 (14)  57(4)  0.10 | 23 (12)  43 (3)  0.25 |
| AFP (ng/mL)  <400 (n=46)  ≥400 (n=14)  *P* | 39 (18)  50 (7)  0.47 | 46 (21)  57 (8)  0.45 | 26 (12)  57 (8)  0.03 | 20 (9)  64 (9)  < 0.01 | 7 (3)  86 (12)  < 0.01 |
| Maximal tumor size (cm)  <5 (n=42)  ≥5 (n=18)  *P* | 45 (19)  33 (6)  0.39 | 55 (23)  33 (6)  0.13 | 26 (11)  50 (9)  0.07 | 33 (14)  22 (4)  0.39 | 26 (11)  22 (4)  0.75 |
| Background liver status  With cirrhosis (n=40)  Without cirrhosis (n=20)  *P* | 43 (17)  40 (8)  0.85 | 48 (19)  50 (10)  0.85 | 30 (12)  40 (8)  0.44 | 35 (14)  20 (4)  0.23 | 30 (12)  15 (3)  0.21 |
| Capsule formation  + (n=35)  - (n=25)  *P* | 43 (15)  40 (10)  0.82 | 49 (17)  48 (12)  0.97 | 37 (13)  28 (7)  0.46 | 37 (13)  20 (5)  0.15 | 26 (9)  24 (6)  0.88 |

*P* values represent the results of the Chi-square test.
